# Iguratimod, an allosteric inhibitor of macrophage migration inhibitory factor (MIF), prevents mortality and oxidative stress in a murine model of acetaminophen overdose

**DOI:** 10.1186/s10020-024-00803-0

**Published:** 2024-03-27

**Authors:** Joshua Bloom, Georgios Pantouris, Mingzhu He, Bayan Aljabari, Lopa Mishra, Ramu Manjula, Andrew Parkins, Elias J. Lolis, Yousef Al-Abed

**Affiliations:** 1 Zucker School of Medicine at Hofstra/Northwell, Hempstead, NY USA; 2 Center for Molecular Innovation, The Feinstein Institutes for Medical Research, Manhasset, NY USA; 3 Department of Emergency Medicine, Warren Alpert Medical School of Brown University, Providence, RI USA; 4 Department of Pharmacology, Yale University School of Medicine, New Haven, CT USA; 5 Department of Chemistry, University of the Pacific, Stockton, CA USA; 6 Institute for Bioelectronic Medicine, The Feinstein Institutes for Medical Research, Manhasset, NY USA; 7 Cold Spring Harbor Laboratory, Cold Spring Harbor, NY USA

**Keywords:** MIF, Iguratimod, Acetaminophen, Enzyme, Drug screening, Inflammation, Drug discovery, Toxicology

## Abstract

**Background:**

Macrophage migration inhibitory factor (MIF) is a pleiotropic cytokine that has been implicated in multiple inflammatory and non-inflammatory diseases, including liver injury induced by acetaminophen (APAP) overdose. Multiple small molecule inhibitors of MIF have been described, including the clinically available anti-rheumatic drug T-614 (iguratimod); however, this drug’s mode of inhibition has not been fully investigated.

**Methods:**

We conducted in vitro testing including kinetic analysis and protein crystallography to elucidate the interactions between MIF and T-614. We also performed in vivo experiments testing the efficacy of T-614 in a murine model of acetaminophen toxicity. We analyzed survival in lethal APAP overdose with and without T-614 and using two different dosing schedules of T-614. We also examined MIF and MIF inhibition effects on hepatic hydrogen peroxide (H_2_O_2_) as a surrogate of oxidative stress in non-lethal APAP overdose.

**Results:**

Kinetic analysis was consistent with a non-competitive type of inhibition and an inhibition constant (K_i_) value of 16 µM. Crystallographic analysis revealed that T-614 binds outside of the tautomerase active site of the MIF trimer, with only the mesyl group of the molecule entering the active site pocket. T-614 improved survival in lethal APAP overdose when given prophylactically, but this protection was not observed when the drug was administered late (6 h after APAP). T-614 also decreased hepatic hydrogen peroxide concentrations during non-lethal APAP overdose in a MIF-dependent fashion.

**Conclusions:**

T-614 is an allosteric inhibitor of MIF that prevented death and decreased hepatic hydrogen peroxide concentrations when given prophylactically in a murine model of acetaminophen overdose. Further studies are needed to elucidate the mechanistic role of MIF in APAP toxicity.

**Supplementary Information:**

The online version contains supplementary material available at 10.1186/s10020-024-00803-0.

## Background

Macrophage migration inhibitory factor (MIF) is a pleiotropic cytokine expressed broadly in multiple cell types (Calandra and Roger 2003). MIF has intrinsic enzymatic activities as both a tautomerase and thiol-protein oxidoreductase (Rosengren et al. 1996; Kleemann et al. 1998). These separate and likely unrelated enzymatic sites are biologically relevant and have been used to guide the development of anti-MIF therapeutics (Pantouris et al. 2015; Thiele et al. 2015). A myriad of anti-MIF small molecules have been identified by our group and others, including compound T-614 (iguratimod), a disease-modifying anti-rheumatic drug approved for clinical use in Japan (Dios et al. 2002; Lubetsky 2002; Al-Abed 2005; Bloom et al. 2016a). Although T-614 is able to inhibit MIF keto-enol tautomerase activity as well as multiple biologically relevant functions, its binding mode and K_i_ have not been described.

Acetaminophen (APAP) overdose is the most common cause of acute liver failure in the United States and causes thousands of hospitalizations and hundreds of deaths yearly (Yoon et al. 2016; Gummin et al. 2022). When taken therapeutically APAP is mostly (∼ 90%) subjected to Phase II metabolism in the liver, resulting in glucoronidated and sulfonated products that can be excreted renally (McGill and Jaeschke 2013). The remainder is oxidized by cytochrome P450 (CYP450) 2E1 to *N*-acetyl-*p*-benzoquinone imine (NAPQI), a reactive metabolite (Dahlin et al. 1984). At therapeutic doses of APAP, the NAPQI produced this way is further metabolized by glutathionylation to form a conjugate that can be excreted via biliary and renal routes (Wong et al. 1981). In overdose, glutathione depletion allows the metabolite NAPQI to cause cell injury and death via formation of reactive oxygen and nitrogen species and the generation of APAP-protein adducts (Blair et al. 1980; Streeter et al. 1984). The only antidote currently available is acetylcysteine, which replenishes hepatic glutathione (Prescott et al. 1977; Yoon et al. 2016). However, it has been proposed to repurpose 4-methylpyrazole (fomepizole) as a therapeutic adjunct given its ability to inhibit CYP450 2E1 and c-Jun n-terminal kinase (JNK) (Akakpo et al. 2019; Link et al. 2022).

MIF is highly expressed in hepatocytes and has been shown to promote liver injury from ethanol and carbon tetrachloride via promoting the release of inflammatory factors, such as tumor necrosis factor alpha (TNFα) and monocyte chemotactic protein 1 (MCP-1) (Barnes et al. 2013; Xie et al. 2016). MIF has also been associated with APAP-induced liver injury: MIF is released by hepatocytes in the first few hours after APAP intoxication, and acetaminophen overdose patients have higher MIF concentrations than non-overdose controls (Bourdi et al. 2002; Bloom et al. 2022). MIF-knockout (KO) animals were protected from both injury and lethality, although APAP-protein adduct formation was not different between the two backgrounds (Bourdi et al. 2002). The MIF inhibitor ISO-1 similarly attenuates inflammation and liver injury in this model (Al-Abed 2005; Ohkawara et al. 2021).

MIF-NAPQI interactions have been investigated previously. NAPQI covalently binds MIF at Pro-1, a relatively basic residue important for MIF’s tautomerase activity (Lubetsky et al. 1999; Senter et al. 2002). However, an attempt to crystallize the MIF-NAPQI complex revealed an APAP dimer, bi-acetaminophen (bi-APAP), and the electron density of the NAPQI adduct was not found (Crichlow et al. 2009). Bi-APAP has been detected in the sera and urine of APAP-intoxicated mice (Chen et al. 2008; Cheng et al. 2009) as well as culture supernatants of APAP-treated primary human hepatocytes (Jetten et al. 2016).

In this study we investigate T-614 as a MIF inhibitor with kinetic studies and X-ray crystallography, revealing a novel binding mode compared to other inhibitors of the tautomerase catalytic activity (e.g., ISO-1). We also apply T-614 to a murine model of lethal APAP overdose, with data suggesting that T-614 prevents lethality when administered as a pretreatment in experimental APAP toxicity. We assess the effects of MIF deletion and inhibition with T-614 on hepatic oxidative stress after APAP overdose. Our data confirms that MIF is involved in APAP toxicity and identifies T-614–a clinically available drug–as another therapeutic agent that can can be investigated in this disease context.

## Methods

### Reagents

Acetaminophen (APAP) and guanidine hydrochloride (Gdn-HCl) were purchased from Sigma-Aldrich (St. Louis, MO). N-acetyl-p-benzoquinoneimine (NAPQI), acetaminophen dimer (bi-APAP), and acetaminophen-glutathione adduct (APAP-GSH) were purchased from Toronto Research Chemicals (Toronto, ON). NAPQI solid was maintained at -80ºC for up to six months, and a 10mM dimethyl sulfoxide (DMSO) stock solution was evacuated under nitrogen gas and stored in -80ºC for up to two weeks. Solid bi-APAP and APAP-GSH were stored at -20ºC for up to one year and solubilized in methanol for chromatography. Iguratimod (T-614) was purchased from Ontario Chemical (Guelph, ON) and stored at -20ºC. Solutions were prepared fresh in alkali (final pH 7.8) as previously described (Sawada et al. 2000) for all studies. We obtained 4-hydroxyphenylpyruvate (4-HPP) from TCI Chemicals (Portland, OR) and prepared it in pH 6.2 the day before experiments.

### Protein expression and purification

Human MIF was expressed and purified following a previously established protocol (Lubetsky 2002; Parkins et al. 2023a). Briefly, a pET-11b plasmid encoding MIF was transformed into BL21(DE3) competent cells (Agilent Technologies - Cat# 200,131) using the heat shock method. The cells were grown at 37˚C, in Luria Broth (LB) containing 100 µg/mL ampicillin, until the solution’s optical density at 600 nm (O.D_600_) became 0.6–0.8. Protein expression was initiated by the addition of 1 mM isopropylthio-β-galactoside (IPTG), at 37˚C, and after four hours the cells were collected and centrifuged. Cells containing MIF were lysed by sonication in 20 mM Tris HCl, 20 mM NaCl, pH 7.4 and loaded onto on Q-Sepharose and SP ion exchange chromatography columns in series. The protein did not bind to either column and eluted in the flow-through with ∼95% purity. MIF impurities were removed by size exclusion chromatography using a 16/60 Superdex 75 column (Cytiva, Marlborough, MA). The running buffer for this step was also 20 mM Tris HCl, 20 mM NaCl, pH 7.4. The final concentration of MIF was determined by the bicinchoninic acid (BCA) protein assay. Sterile recombinant MIF was maintained as 0.5-1 mg/mL stock solutions in 20 mM Tris, 20–150 mM NaCl, pH 7.4 (human) or pH 6.8 (mouse) for up to six months with no appreciable loss of activity by tautomerase assay.

### MIF enzyme assays and kinetic analyses

Dopachrome and 4-HPP enzymatic activities of MIF were assayed as described previously (Dios et al. 2002; Parkins et al. 2023b). For dopachrome tautomerase inhibition, compounds were prepared as 100 mM stock solutions in DMSO, added to a 1.5mL cuvette (Crystalgen Inc. Commack, NY) containing 1 µg/mL MIF in PBS pH 7.4, and mixed thoroughly. Dopachrome substrate was added and the solution monitored at 475 nm for 20 s to measure activity. For 4-HPP tautomerase inhibition, a 30 mM stock of 4-HPP was prepared in 0.5 M ammonium acetate pH 6.2 and incubated overnight (∼16 h) at room temperature while rocking. Then, 4-HPP was added to a 96-well microplate in a gradient of 0–2 mM final concentration, followed by the borate solution (pH 6.2) at a working concentration of 0.42 M. Inhibitor was added at a final concentration ranging from 0 to 50 µM in 1% DMSO. The reaction was then initiated by the addition of MIF at a final concentration of 50 nM, bringing the total well volume to 150 µL. Conversion of keto-HPP to enol-HPP was measured through the formation of the enol-HPP/borate complex (ε_306_ = 11,400 M^− 1^ cm^− 1^) at 306 nm. Readings were taken every 10 s for a total period of 180 s using a Tecan Infinite M-Plex microplate reader (TECAN). The experiments were carried out in triplicate and the data was analyzed in GraphPad Prism 9.

### Crystallization of MIF-T-614 complex and structure determination

Crystallization of MIF-T-614 complex was performed by the vapor diffusion method in 24-well hanging drop trays as reported previously (Pantouris et al. 2014). Freshly purified MIF (∼18 mg/mL) was mixed with T-614 at 1:3 protein-inhibitor molar ratio and incubated overnight at 4˚C. The next day, after removing precipitation by centrifugation, the MIF-T-614 complex was mixed with the well solution containing 20 mM Tris HCl pH 7.5, 2 M (NH_4_)_2_SO_4_, and 3% 2-propanol. Protein and well solutions were mixed at different volume ratios, retaining the total drop volume at 4 µL. Crystallization trays were stored at 20˚C and checked on a regular basis. Crystals were formed and grew to their full size within two weeks. For diffraction screening and data set collection, crystals were flash frozen in the mother liquor enriched with 28% glycerol (cryoprotectant). A complete data set was collected at the Macromolecular Crystallographic Facility of Yale School of Medicine using Rigaku Pilatus 200 K Detector with a Rigaku 007 rotating copper anode X-ray generator (wavelength = 1.5418 Å) at a temperature of 100 K. Integration and scaling was performed in HKL-2000 (Otwinowski and Minor 1997), while the solution was obtained by molecular replacement using PHASER (McCoy et al. 2007). The initial model of MIF-T-614 complex was refined using Refmac (Winn et al. 2003) and COOT (Emsley et al. 2010) and visualized with PyMOL (Delano 2002). The supporting files for T-614 (PDB and CIF) were produced by PRODRG (Schüttelkopf and Aalten 2004). Superposition of the MIF-T-614 crystal onto the corresponding structure of wild-type MIF (WT MIF) (PDB entry:3DJH) was performed in SUPERPOSE, a CCP4 supported program (Winn et al. 2011). The crystallographic table of MIF-T-614 is provided in Suppl. Table 1 and the structure was deposited in PDB under the accession code 8SPN.

### Animal experiments

The Institutional Animal Care and Use Committee at the Feinstein Institute for Medical Research reviewed and approved all animal protocols prior to initiation of experiments. Male C57BL/6 NCr mice were purchased from Charles River Laboratories (Stone Ridge, NY) and acclimated in our facility for at least seven days. MIF KO animals were maintained on a C57BL/6 NCr background from the source colony described by Bozza and colleagues (Bozza et al. 1999). All animals were maintained on PicoLab Rodent Diet 20 from LabDiet (St. Louis, MO) and used between the ages of 8–12 weeks. Death was not an endpoint, and when animals were deemed moribund, they were euthanized.

### APAP-induced hepatotoxicity

For all experiments, animals were fasted for 16 h by transferring them to clean cages that never contained food prior to dosing with APAP. APAP was administered by intraperitoneal injection of a 15 mg/mL solution in warm 0.9% saline (Hospira, Lake Forest, IL). Injection volumes were adjusted to mouse weight. Volumes up to 0.8 mL were well tolerated. After APAP administration, food was provided *ad libitum*. For survival experiments, animals were dosed with 420 mg/kg APAP and monitored for two weeks; when applicable, T-614 (20 mg/kg) was administered intraperitoneally 1 h pre-APAP, 6 h post-APAP, and once daily in the morning for four days afterward. For acute toxicity experiments, animals were given a non-lethal dose of APAP (300 mg/kg) and euthanized at four hours post-APAP by CO_2_ asphyxiation with cervical dislocation. Blood and liver were harvested for analysis. When applicable, T-614 (20 mg/kg) was administered intraperitoneally 1 h pre-APAP and 1 h post-APAP. Blood was collected by cardiac puncture and allowed to clot for 20 min at room temperature and 20 min at 4ºC. Sera were isolated by centrifugation at 300xg for 10 min and stored at -20ºC for further analysis. Livers were mobilized and divided into major lobes, and median lobes were processed for H_2_O_2_ studies. For urine collection experiments, animals were dosed with 400 mg/kg APAP and left undisturbed for 24 h, after which urine was collected by the “single-animal method” as previously described (Kurien and Scofield 1999). Collections were done on parafilm, and 2–3 collections were performed within a 1-hour period. Urine proteins were denatured by 1:1 addition of acetonitrile (Fisher), flash frozen in liquid nitrogen, and lyophilized to dryness. Dried samples were stored at -20ºC until analysis. Liver samples subjected to HPLC analysis were similarly denatured, flash frozen, lyophilized, and stored at -20ºC until analysis.

Serum levels of alanine transaminase (ALT) were determined using kits purchased from Bioo Scientific (Austin, TX). Serum concentrations of cytokines were determined using DuoSet ELISA kits from R&D Systems (Minneapolis, MN). Liver H_2_O_2_ content was determined using a Hydrogen Peroxide Assay Kit from Abcam (Cambridge, United Kingdom). For this application, livers were homogenized by 15 passes in a Dounce homogenizer and deproteinized with perchloric acid as per the manufacturer’s recommendation, and assays were performed on the same day as tissue isolation.

### Statistics

In this study, we attempted to display individual data point values when feasible. Raw data and calculations can be made available on request. Statistical methods are cited when applicable, and calculations were done using GraphPad Prism (San Diego, CA).

## Results

**Fig. 1:**
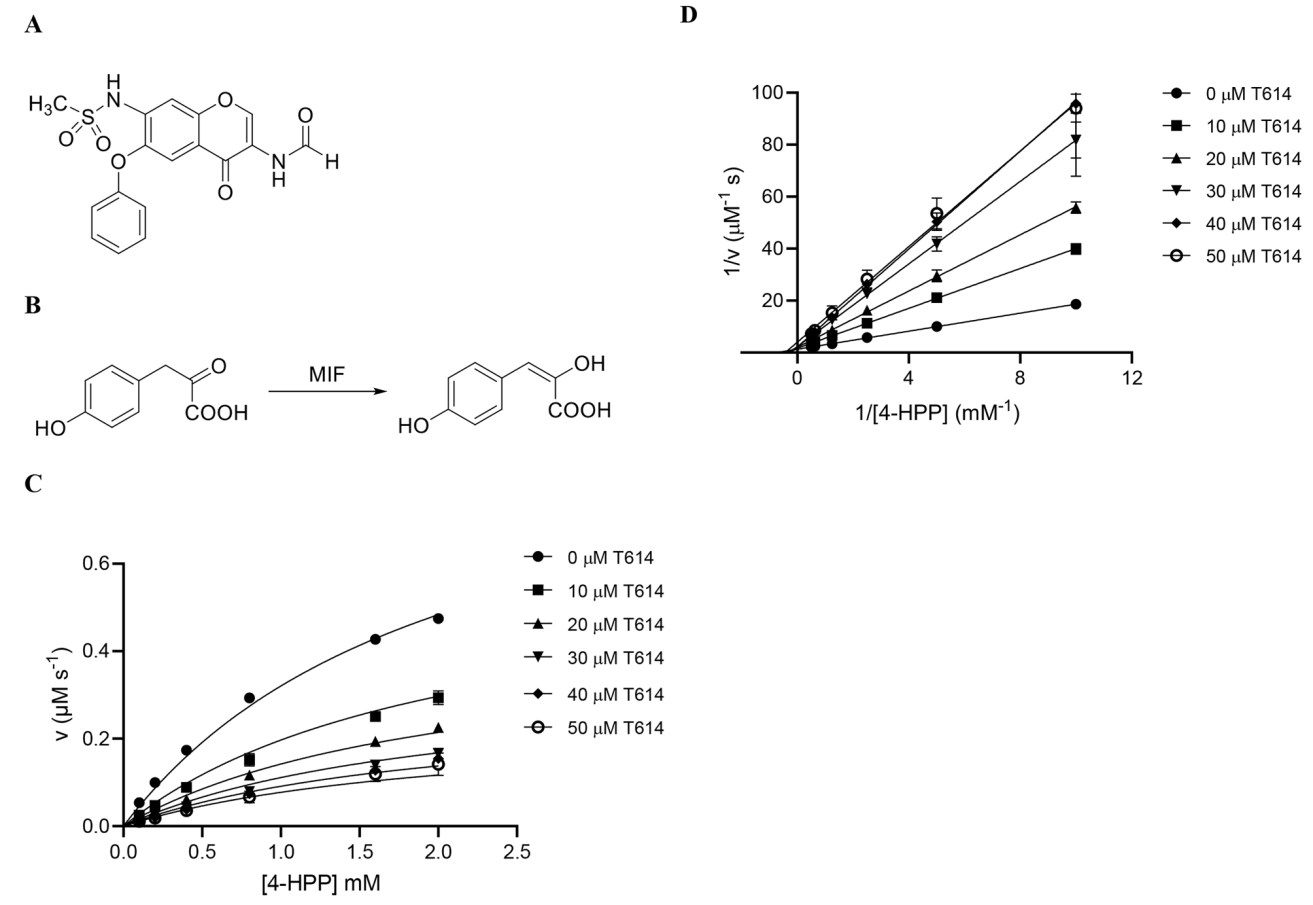
Kinetic analysis of MIF-T-614 complex. Chemical structures of (**A**) the MIF inhibitor T-614 and (**B**) the substrate 4-HPP for MIF-induced keto-enol tautomerization. (**C**) The Michaelis-Menten and (**D**) Lineweaver-Burk plots demonstrate that the inhibitor binds MIF in a non-competitive fashion. The inhibition potency of T-614 against MIF’s keto-enol tautomerase activity was investigated at concentrations ranging from 0–50 µM and yielded an inhibition constant (K_i_) of 16 µM. Data is expressed as mean ± SD from experiments done in triplicate (*n* = 3). T614 = iguratimod

**T-614 is a non-competitive inhibitor of MIF with a novel binding motif.** To determine the K_i_ value of T-614, we performed a kinetic experiment using 4-HPP as the model substrate (Fig. 1A-B). The Michaelis-Menten (Fig. 1C) and Lineweaver-Burk plots (Fig. 1D) demonstrate that T614 binds MIF as a non-competitive inhibitor with a K_i_ value of 16 µM, which is on a similar scale to the IC_50_ of 6.8 µM (human MIF) with dopachrome substrate as described previously (Bloom et al. 2016a), and the IC_50_ of 5.2 µM we have observed in murine MIF. Of note, the serum C_max_ of T-614 in humans after multiple twice-daily doses of 25 mg tablets is 4.3–4.6 µM depending on age group (Careram (R) [package insert] 2013).

**Fig. 2:**
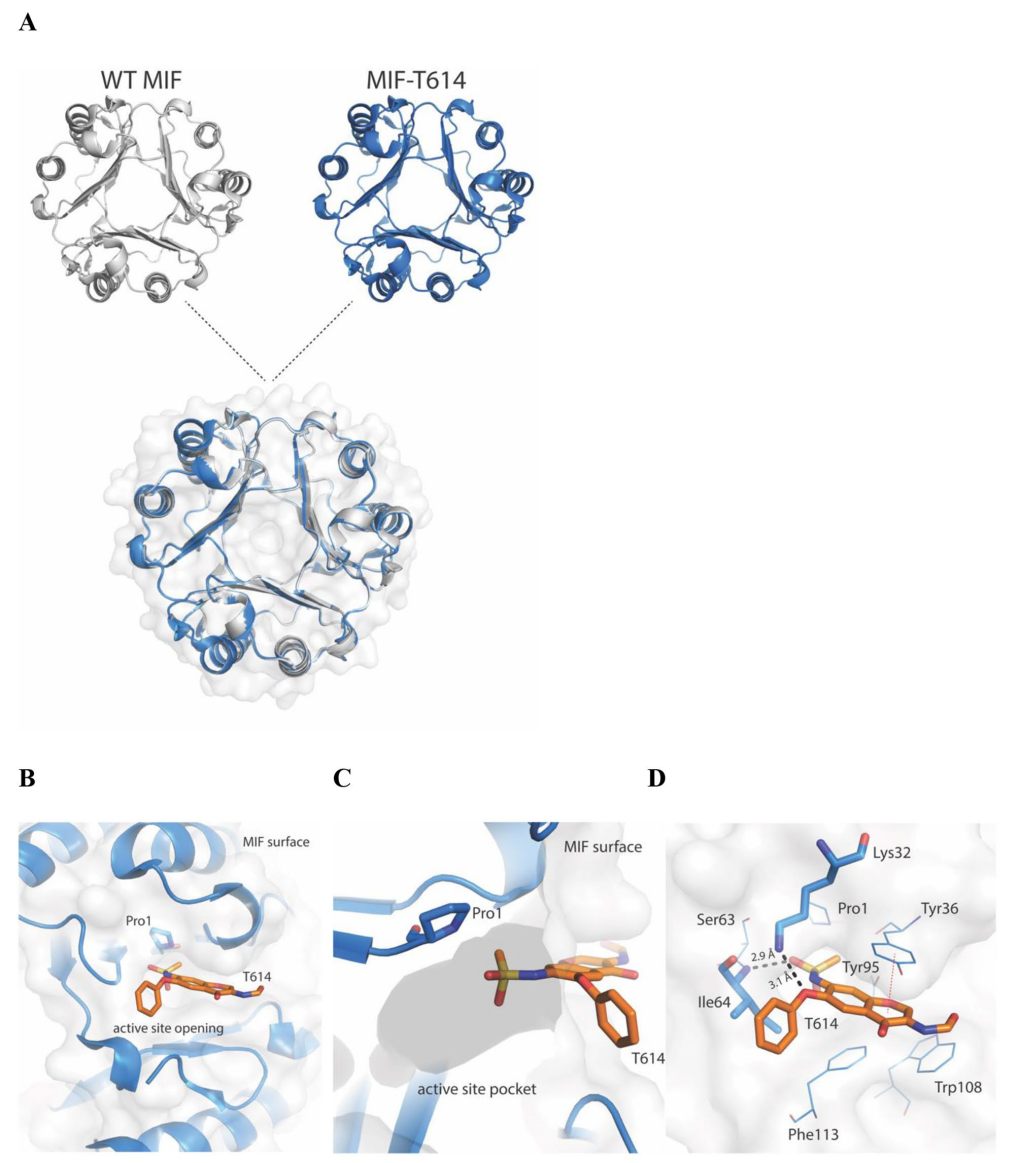
Crystallographic analysis of the MIF-T-614 complex. (**A**) Alignment of WT MIF (grey cartoon) onto MIF-T-614 (blue cartoon) demonstrates high superposition agreement between the two crystal structures. (**B**) Analysis of the binding motif of T-614 (orange sticks) showed that the inhibitor predominantly binds on the surface of MIF, blocking the active site opening. For reference, the position of the catalytically active Pro-1 (blue sticks) is provided. (**C)** Only a small chemical moiety of bound T-614 penetrates the active site. The active site of MIF is illustrated as a dark grey shadow. (**D**) Stabilizing forces between T-614 and MIF. T-614 forms hydrogen bonds (Lys-32 and Ile-64, black dashed lines, residues shown as sticks), van der Waals interactions (Pro-1, Lys-32, Tyr-36, Ser-63, Ile-64 Trp-108 A and Phe-113 from subunit C and Tyr-95 from subunit B, black lines), and Pi-Pi interactions (Tyr-36, red dashed line) with MIF residues. T614 = iguratimod

To further explore the binding motif of this inhibitor, we performed MIF-T-614 co-crystallization experiments. The electron density map of T-614 was clearly observed in one of the three MIF subunits (Suppl. Table 1, Suppl. Fig. 1). Alignment of MIF-T-614 crystal structure onto the corresponding structure of WT MIF demonstrated high superposition agreement with a root-mean-square deviation (RMSD) value of 0.17 Å, indicating that binding of T-614 did not cause any major conformational changes (Fig. 2A).

To further elucidate the effect of T-614 binding, we performed structural analysis around the inhibitor’s binding site. Surprisingly, T-614 primarily binds on the surface of MIF blocking the catalytic pocket of the tautomerase active site (Fig. 2B). The mesyl group of T-614 is the only chemical moiety that penetrates the pocket (Fig. 2C). Apart from the van der Waals interactions with Pro-1, Ser-63, Ile-64 from chain C and Tyr-95 from chain B, the mesyl group also forms a hydrogen bond with Ile-64 at 2.9 Å (Fig. 2D). Another hydrogen bond at 3.1 Å was noted between T-614 and Lys-32, while Tyr-36 is located less than 4 Å from T-614 and forms Pi-Pi interactions with the inhibitor (Fig. 2D).

**Fig. 3:**
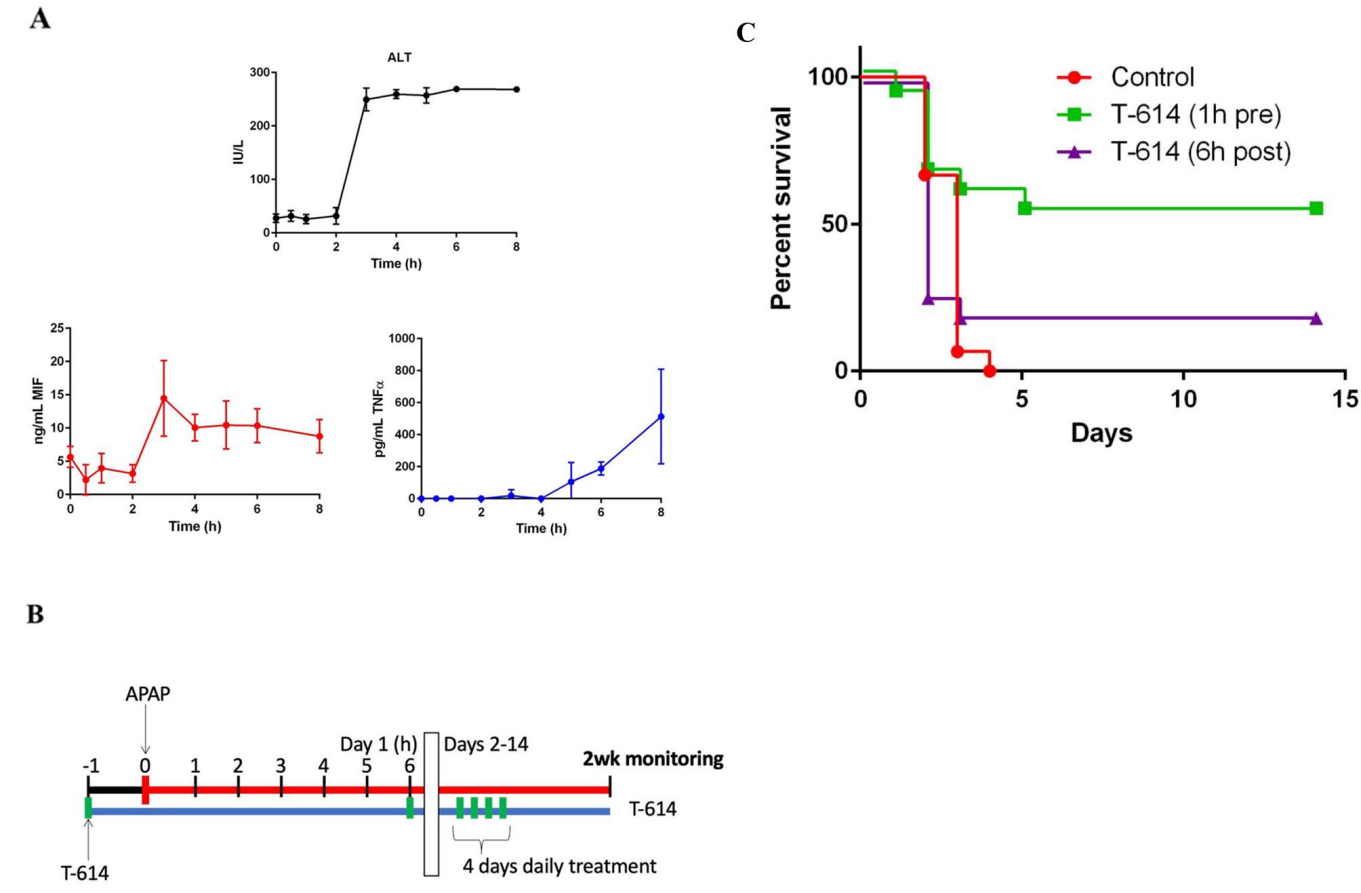
Early administration of T-614 prevents lethality in APAP overdose. (**A**) Serum concentrations of MIF peak with ALT, and prior to TNFα. C57BL/6 mice (*n* = 3–4/group) were given 300 mg/kg APAP intraperitoneally and euthanized at indicated time points for blood collection by cardiac puncture. Results are shown mean ± SD. (**B-C**) C57BL/6 wild-type mice (*n* = 10/group) were given 420 mg/kg APAP intraperitoneally in addition to T-614 vehicle (control) or T-614 initiated as pretreatment (1 h pre) or post-treatment only (6 h post) as described in Materials and Methods, and were monitored for two weeks; methods and time course are summarized in (**B**), and results are shown in (**C**). Death was not an endpoint for this experiment. A log-rank test was used to determine statistical significance: Control vs. T-614 (1 h pre), **p < 0.01; Control vs. T-614 (6 h post), *p* > 0.05. T-614 = iguratimod

**T-614 pretreatment attenuates lethality in a model of lethal APAP overdose.** We observed that animals given a non-lethal overdose of APAP (300 mg/kg) showed a peak in MIF concentrations roughly coincident with ALT, and preceding elevations in another pro-inflammatory cytokine, TNFα (Fig. 3A), which is consistent with the findings of Bourdi and colleagues (Bourdi et al. 2002). Based on this we designed an experiment comparing T-614 treatment prior to and after this peak. Control animals dosed with 420 mg/kg intraperitoneal APAP had 100% mortality. T-614 treatment initiated prior to APAP overdose yielded a statistically significant improvement in survival (*p* < 0.01 by log-rank test), but treatment initiated 6 h after overdose was not significantly different from control (Fig. 3B-C).

**Fig. 4:**
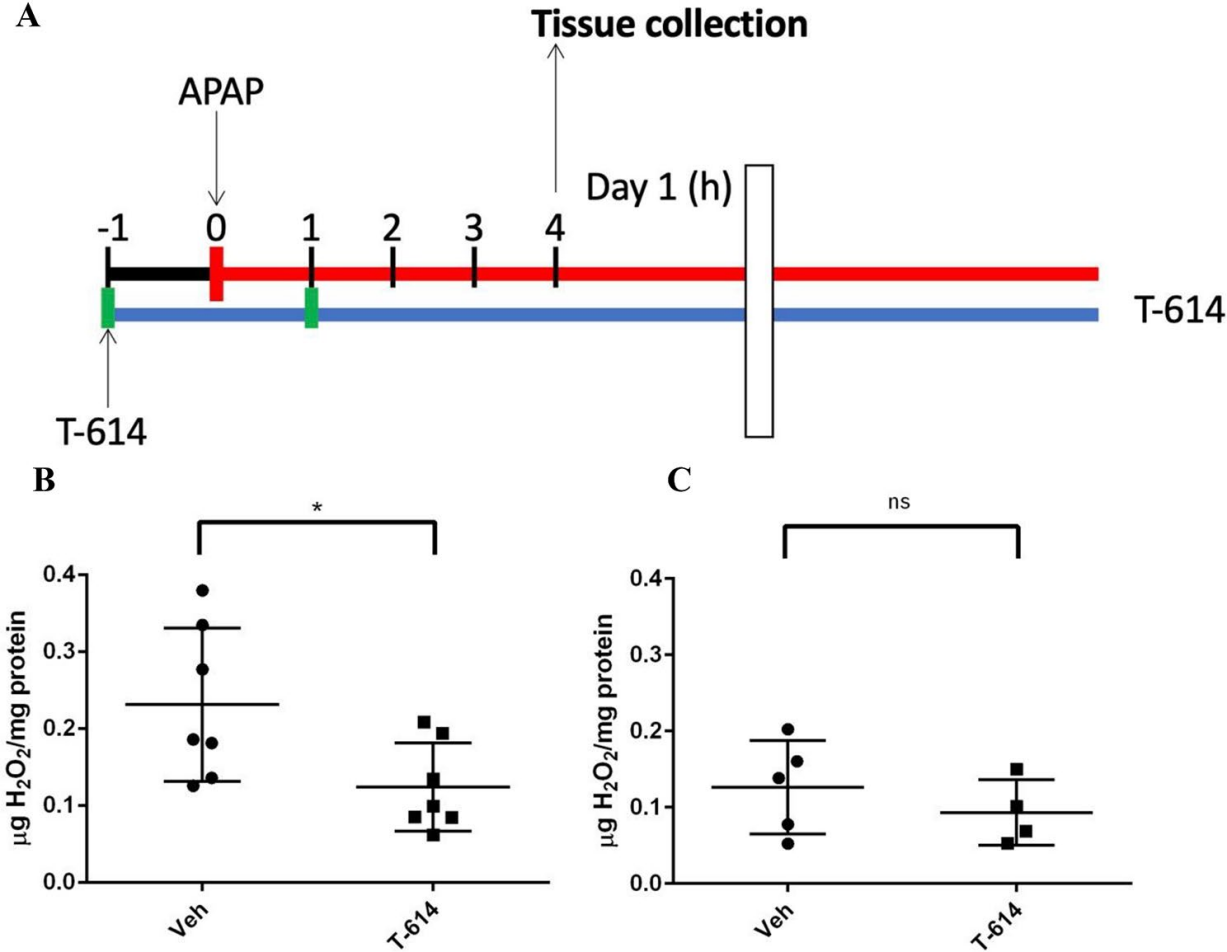
T-614 prevents oxidative stress in APAP overdose. (**A**) Experimental summary. (**B**) C57BL/6 wild-type and (**C**) MIF KO mice (*n* = 4–7/group) were given 300/kg APAP intraperitoneally in addition to doses of intraperitoneal T-614 or vehicle (1 h pre- and post-APAP) and euthanized at 4 h for tissue collection and H_2_O_2_ assay as described in Materials and Methods. Results are shown as mean ± SD and were analyzed using unpaired t-tests with one-tailed p-values given: *, *p* < 0.05. T614 = iguratimod

**T-614 prevents oxidative stress in the APAP model.** Cellular toxicity in APAP overdose is partly mediated by reactive oxygen species (ROS) generated after the production of NAPQI in hepatocytes. Previous studies have shown that reactions between MIF and NAPQI can generate an MIF-NAPQI adduct or an APAP dimer, bi-APAP (Senter et al. 2002; Crichlow et al. 2009). We have also observed that bi-APAP and APAP can be generated when these molecules are co-incubated, and excretion of bi-APAP in murine APAP toxicity appears to be MIF dependent (Suppl. Figures 2–4). Given the possibility that MIF could be involved in oxidation-reduction processes during APAP toxicity, we wanted to pursue a potential effect of MIF on oxidative stress in this model (Fig. 4A). Hydrogen peroxide is a byproduct of ROS generation in vivo, and can be directly measured as a surrogate of oxidative stress in tissue. T-614-treated WT mice given a non-lethal overdose of APAP had significantly less H_2_O_2_ detected in hepatic tissue (Fig. 4B). MIF KO animals treated under the same conditions showed a similarly significant decrease in hepatic tissue H_2_O_2_ (*p* < 0.05 for non-T-614 treated WT versus MIF KO), and no additional effect of T-614 was seen in MIF KO animals (Fig. 4C), suggesting T-614 effects on this readout may be MIF-specific.

## Discussion

T-614 is an effective disease-modifying antirheumatic drug currently in clinical use in Japan. Initial studies suggested its mechanism of action may operate via interactions with cyclooxygenase-2 or effects on translocation of nuclear factor kappa B. Our group recently established that it functions as a MIF inhibitor as well, and this bioactivity could explain some of its anti-inflammatory actions (Tanaka et al. 1995; Aikawa et al. 2002; Bloom et al. 2016a). Given MIF is a broadly expressed cytokine with putative roles in multiple disease states, the application of a clinically available drug that can modulate MIF activity could have important implications, and there is a need to explore the therapeutic potential of repurposing this drug (Bozza et al. 1995; Onodera et al. 2000; Jong et al. 2001). Our findings here confirm that T-614 not only interacts with the MIF trimer, but also acts as an allosteric inhibitor with a novel binding mode. Tautomerase inhibition is mediated by steric blockade of the active site. Occupancy in this region may also disrupt MIF interactions with its putative receptor CD74, and subsequent pro-inflammatory effects, although further studies would be needed to confirm this (Leng et al. 2003; Pantouris et al. 2014). The structural interactions between MIF and CD74 have been mapped previously, and T-614 interacts with multiple residues shown to have a key role in the MIF-induced activation of CD74 (Pantouris et al. 2015).

MIF has previously been studied in APAP toxicity, and the pathophysiology of its interaction with NAPQI has been poorly elucidated. Coincubation of NAPQI and MIF appeared to produce a MIF-NAPQI adduct at the nucleophilic Pro-1 based on mass spectrometry analysis, but a P1S mutant MIF did not generate an adduct (Senter et al. 2002). A crystallography study did not show this adduct, and instead revealed a noncovalently bound molecule of bi-APAP at the MIF active site (Crichlow et al. 2009). Our comparatively simple chromatography experiments similarly revealed a mixture of products after MIF-NAPQI co-incubation, and it seems likely that both the MIF-NAPQI adduct and bi-APAP can be generated in this reaction, among other potential products. Bi-APAP may not be a direct enzymatic product, but rather a side product formed by comproportionation between NAPQI and residual APAP, which has been described previously (Potter and Hinson 1986). Studies of relative affinity of NAPQI for APAP and glutathione suggest that dimer formation would be inhibited in the presence of glutathione, which has a much higher second-order rate constant for NAPQI at pH 6.5 (1 × 10^4^M^− 1^sec^− 1^) than APAP (33M^− 1^sec^− 1^) (Potter and Hinson 1986). In our experimental model of NAPQI incubation, bi-APAP peaks could not be detected when glutathione was present (Suppl. Figure 4A). It is possible that in the absence of glutathione–for example, during the glutathione depletion observed in APAP overdose–dimer formation is permitted via comproportionation reactions between APAP and NAPQI that produce N-acetyl-p-benzosemiquinone imine (NAPSQI), which can react further to produce APAP polymers such as bi-APAP. MIF appears to facilitate or permit this process, since a MIF KO animal excreted significantly less bi-APAP, although whether this effect is attributable to a direct interaction between MIF and NAPQI or another mechanism is unclear. Partial ablation of tautomerase activity by denaturation of MIF with Gdn-HCl did not affect the production of bi-APAP after coincubation (Suppl. Figure 4B). Of note, MIF can also act as a thiol-protein oxidoreductase based on a CXXC motif around Cys-56 and Cys-59, and this motif and activity appear important for MIF interactions with intracellular binding partners (Kleemann et al. 1998; Bloom et al. 2016b); it is unknown at this time whether this motif influences MIF effects on APAP toxicity.

Our findings suggest that MIF inhibition with T-614 can prevent APAP toxicity: T-614-treated animals had less hydrogen peroxide present in liver tissue, and no additional effect of T-614 was seen in MIF KO mice. As we do not document baseline hydrogen peroxide levels in WT mice, it is unclear if T-614 treatment restored these levels to an untreated baseline. It is possible that the extent of protection from MIF genetic deletion was such that any additional non-MIF-related protective effect of T-614 would not be observable. Additionally, non-MIF related effects are possible, such as T-614 inhibition of CYP450. However, our results suggest that the effects of T-614 could be MIF-specific. Based on our observation that lethality in the APAP model was only attenuated by early administration of T-614, MIF’s pathologic role in murine APAP toxicity is expected to occur earlier than six hours after APAP administration. This accords with observations by our group and others of early peaks in serum MIF concentrations after APAP overdose (Bourdi et al. 2002). Of note, our experiments demonstrated a relatively milder level of toxicity (based on lethality and ALT release) per APAP mg/kg dosing compared to Bourdi’s study or more recent studies using this mouse background (Duan et al. 2016). Although our experimental conditions differed slightly from these studies, this is an important limitation to our findings.

Unlike conventional cytokines, MIF is expressed at baseline and is stored in cytoplasmic pools for release in response to stimuli; humans also have detectable MIF in plasma at baseline (Bacher et al. 1997; Kudrin et al. 2006). The early phase of APAP toxicity involves NAPQI generation and cellular toxicity via generation of reactive species and protein adduct formation. Subsequent sterile inflammation is marked by the release of conventional cytokines such as TNFα, which we observed beginning at 4 h. Since we only compared treatment with T-614 prior to APAP and 6 h after APAP administration, we are unable to determine by time course alone whether the benefits of MIF inhibition occurred during the NAPQI/reactive products phase or the inflammatory phase, which is a limitation of our study. Other important limitations of our study include a lack of liver histology to confirm liver injury, and a lack of ALT measurement at all doses and time points in our models. In the absence of this information, we cannot recommend clinical application of T-614 in APAP toxicity as yet. Future studies could help resolve this question by testing anti-MIF therapy at more acute time points (before 6 h) with more extensive monitoring of liver histology and transaminases, or probing other biomarkers of APAP toxicity (Davern et al. 2006); comparison with other known and proposed antidotes for APAP toxicity (acetylcysteine, fomepizole) would also be useful. Of interest, prior studies of MIF KO showed no effect on APAP-protein adducts, but inhibition with ISO-1 resulted in increased measured glutathione stores in liver tissue from APAP-intoxicated mice, which would seem to be contradictory (Bourdi et al. 2002; Ohkawara et al. 2021). Early studies of MIF suggested possible enzymatic activity as a glutathione-S-transferase, but subsequent experiments did not support this (Blocki et al. 1992; Pearson 1994; Mühlhahn et al. 1996; Swope et al. 1998).

Prior investigations have suggested that JNK1 and JNK2 are mechanistically important in the development of hepatocellular toxicity from APAP, and the alcohol dehydrogenase and JNK inhibitor 4-methylpyrazole (fomepizole) is under investigation as potential target for drug repurposing in APAP toxicity (Gunawan et al. 2006; Akakpo et al. 2019). Fomepizole was able to provide hepatoprotection by multiple readouts when dosed as either a pre-treatment or 90 min post-APAP (Akakpo et al. 2018, 2019). MIF is known to influence JNK phosphorylation, although its effects appear to be pleiotropic based on cell type: recombinant MIF stimulated JNK phosphorylation in fibroblasts and T-cell lines but attenuated it in murine hepatocytes and HepG2 cells under lipemic conditions (Lue et al. 2011; Cui et al. 2022). The MIF-JNK relationship may affect or drive MIF-mediated effects in APAP toxicity, and future studies are needed to investigate this.

## Conclusions

We have found that T-614 is an allosteric inhibitor of MIF with a unique binding mode outside the catalytic pocket of the tautomerase active site. Our work also establishes that it can prevent oxidative stress and lethality in a murine model of APAP hepatotoxicity, suggesting that T-614 should be investigated further to determine its therapeutic potential in APAP hepatotoxicity. Further studies are needed to elucidate the molecular mechanisms of MIF and MIF inhibition in this disease process.

### Electronic supplementary material

Below is the link to the electronic supplementary material.

Supplementary Material 1

Supplementary Material 2

Supplementary Material 3

## Data Availability

The crystallographic structure of MIF-T-614 was deposited in Research Collabatory for Structural Bioinformatics Protein Data Bank (RCSB PDB) under the accession code 8SPN.

## Abbreviations

4-HPP: 4-hydroxyphenylpyruvate
ALT: Alanine transaminase
APAP: Acetaminophen
BCA: bicinchoninic acid
Bi-APAP: bi-acetaminophen
CYP450: Cytochrome P450
Cys: Cysteine
DMSO: Dimethyl sulfoxide
Gdn: Guanidinium
GSH: Glutathione
HPLC: High performance liquid chromatography
IPTG: isopropylthio-β-galactoside
JNK: c-Jun n-terminal kinase
KO: Knockout
Ki: Inhibition constant
LB: Luria Broth
MCP-1: Monocyte chemotactic protein 1
MIF: Macrophage migration inhibitory factor
NAPQI: N-acetyl-p-benzoquinoneimine
NAPSQI: N-acetyl-p-benzosemiquinoneimine
PBS: Phosphate-buffered saline
ROS: Reactive oxygen species
T-614: Iguratimod
TBS: Tris-buffered saline
TNFα: Tumor necrosis factor alpha
WT: Wild type
