# Supplementary material for: Iguratimod, an allosteric inhibitor of macrophage migration inhibitory factor (MIF), prevents mortality and oxidative stress in a murine model of acetaminophen overdose
Source: Mol Med. 2024 Mar 27;30:43. doi: 10.1186/s10020-024-00803-0 (PMC10976746; doi:10.1186/s10020-024-00803-0)
Supplement: Supplementary file 1 — Supplementary Material 1 [file 10020_2024_803_MOESM1_ESM.docx]

**Supplemental Table 1:** **Data collection and refinement statistics for MIF-T614.** *T614 = iguratimod.*

|  | MIF-T614 |
| --- | --- |
| **Data collection** |  |
| Space group | P 21 21 21 |
| Cell dimensions |  |
| *a*, *b*, *c* (Å) | 67.68, 67.94, 88.37 |
| α, β, γ (°) | 90.00, 90.00, 90.00 |
| Resolution (Å) | 50.00 - 1.73(1.78-1.73) * |
| *R*_sym_ or *R*_merge_ | 0.047(0.118) |
| *I* / σ*I* | 24.5(9.9) |
| Completeness (%) | 99.5(98.2) |
| Redundancy | 3.9(3.6) |
|  |  |
| **Refinement** |  |
| Resolution (Å) | 42.18-1.73 |
| No. reflections | 40780 |
| *R*_work_ / *R*_free_ | 0.17/0.20 |
| No. atoms |  |
| Protein | 2568 |
| Ligand/ion | 46 |
| Water | 432 |
| *B*-factors |  |
| Protein | 13 |
| Ligand/ion | 23 |
| Water | 29 |
| R.m.s. deviations |  |
| Bond lengths (Å) | 0.027 |
| Bond angles (°) | 2.371 |
|  |  |

* Values in parentheses are for highest-resolution shell.

**ADDITIONAL FILE 1: Methods for Supplemental Figures 1-3**

*MIF-NAPQI Reactions*

MIF-NAPQI co-incubation experiments were conducted in TBS with or without MIF at concentrations ranging from 0.7-1 mg/mL. NAPQI from a stock solution (10 mM in DMSO) was added at a 1:1 or 1:5 molar ratio (MIF:NAPQI) to the reaction mixture and incubated at room temperature for 0-60 minutes prior to injection for high-performance liquid chromatography (HPLC). Human and mouse MIF proteins produced similar results in this assay.

*High-Performance Liquid Chromatography (HPLC) and Mass Spectrometry (MS)*

For analysis of MIF-NAPQI co-incubation experiments, 45 μL samples were injected into a Waters 1825 HPLC system (Waters, Milford, MA) using a 2.6 µm Kinetex C18 column (4.6x150 mm) at ambient temperature. A linear gradient of 5-95% acetonitrile in water/0.05% trifluoroacetic acid (TFA) was used for 15 minutes with a flow rate of 1 mL/min. Peaks were monitored by ultraviolet detection at 254 nm using a photodiode array (PDA) detector. Relative abundances of APAP and bi-APAP were determined by integration of peak areas and comparison to calibration curves generated by preparing pure standards in TBS.

For urine analysis of bi-APAP, we adapted the method of Chen and colleagues (Chen et al. 2008) with some modifications. Briefly, lyophilized samples were reconstituted in acetonitrile, vortexed, and pelleted under high-speed centrifugation for 1 minute. Supernatant (10 μL) was injected and analyzed on an Agilent 6550 QToF coupled to an Agilent 1290 Infinity LC system, with an Agilent Poroshell 120 SB-C18 column (2.7 um, 2.1x50 mm) at 45ºC. A linear gradient of 5-95% acetonitrile in water (0.1% formic acid) was used for 10 min with a flow rate of 0.4 mL/min. Data were analyzed using MassHunter Qualitative Analysis B.06.00 (Agilent Technologies, Santa Clara, CA). Bi-APAP was identified through accurate mass measurements by comparison with pure standards. Peak areas of bi-APAP were integrated and quantitated through the use of a calibration curve. The calibration curve was prepared by adding bi-APAP standards to blank mouse urine, where a linear range of 0.1 pg/μL to 5.0 pg/μL (1 pg to 50 pg on column) of bi-APAP was obtained. The relative abundances of APAP were determined by normalizing single ion counts of bi-APAP to the total ion counts for each sample (single ion counts/total ion counts).

**SUPPLEMENTAL FIGURES**


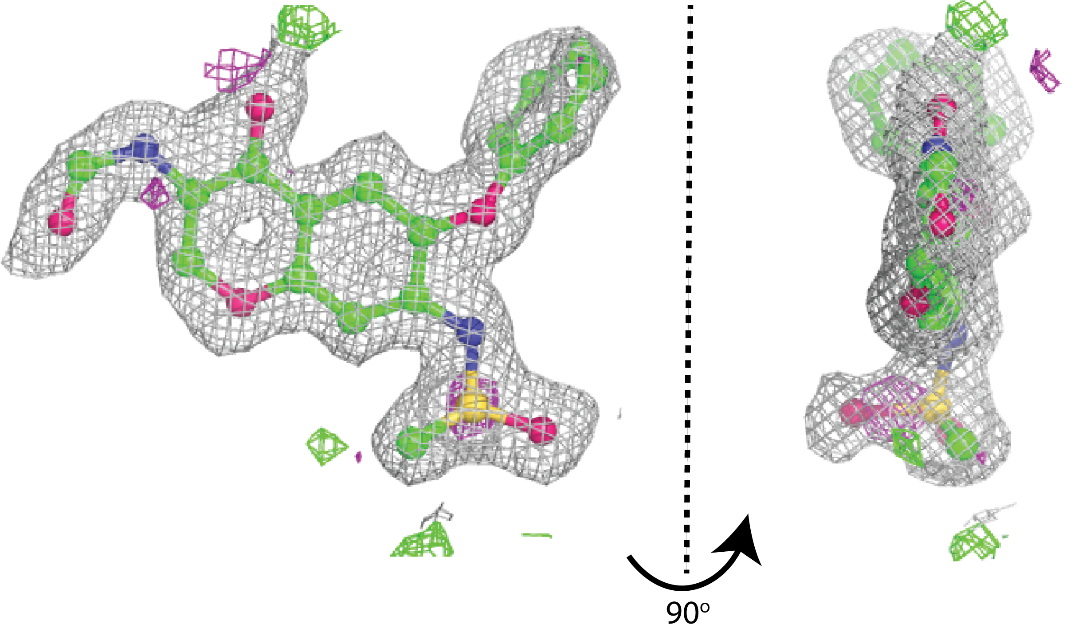


**Supplemental Figure 1: Electron density maps of T614.** *A complete electron density map of T-614 was detected in one of the three MIF subunits. The 2Fo-Fc (grey) and Fo-Fc (purple) maps of T-614 were contoured at 0.7σ and 3σ, respectively. The positive electron density is shown in green.*

**A**


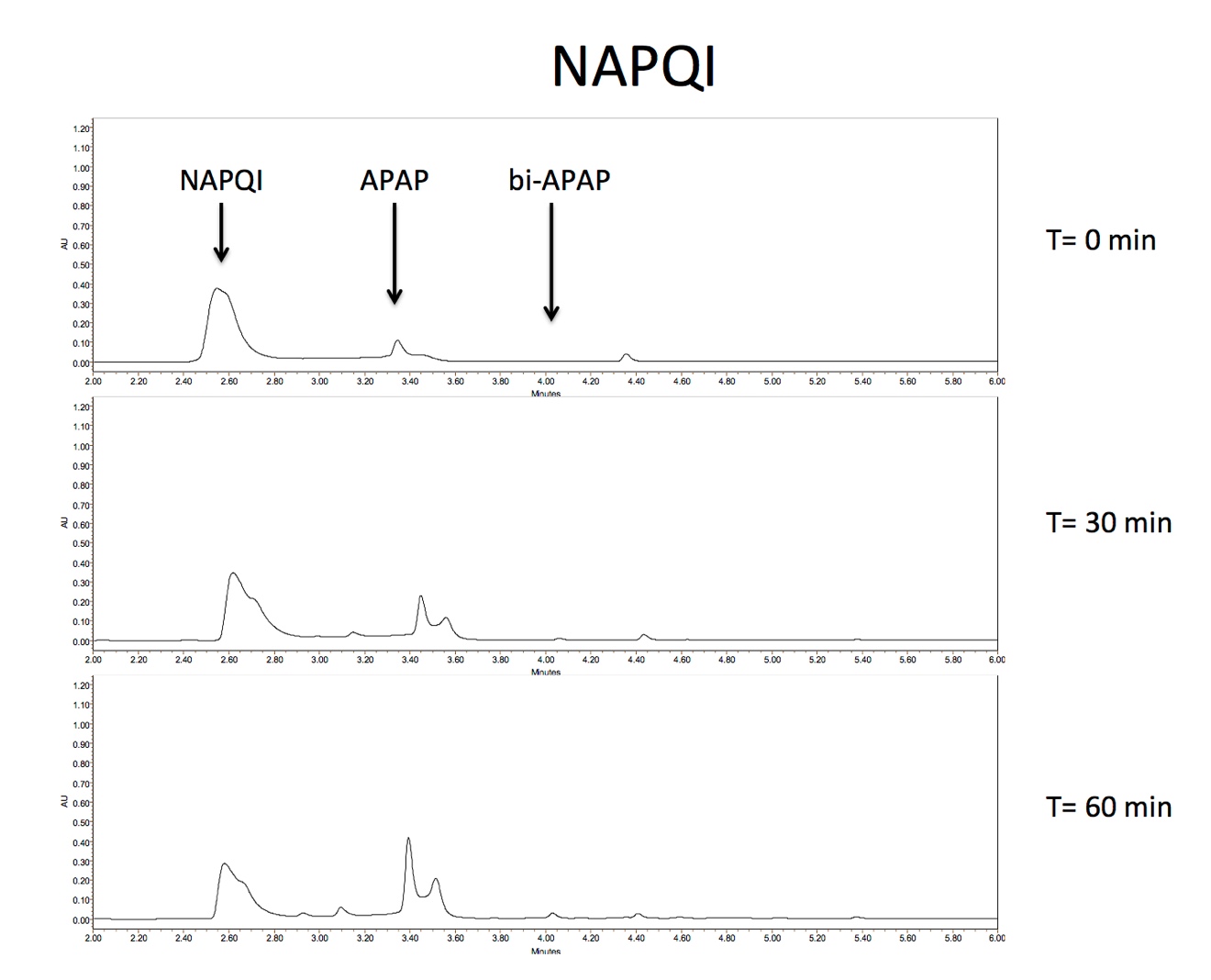


**B**

**
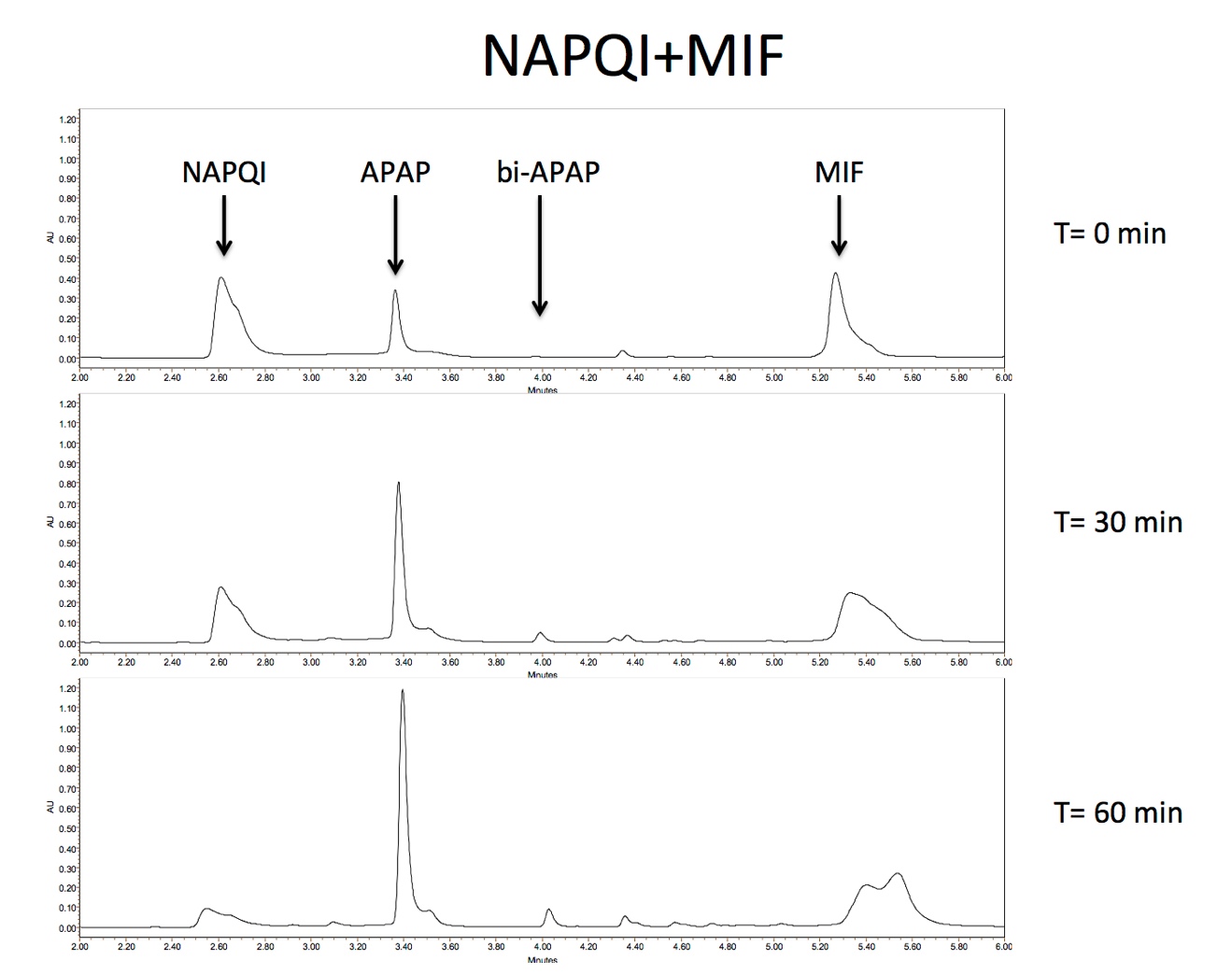
**

**Supplemental Figure 2: Bi-APAP and APAP are generated in vitro when NAPQI is co-incubated with MIF.** *HPLC chromatograms of samples taken from (****A****) NAPQI and (****B****) NAPQI/MIF reaction mixtures at indicated time points as described in Materials and Methods. Peaks corresponding to NAPQI, APAP, bi-APAP, and MIF are labeled where appropriate in the first chromatogram, and 0 and 60 minute chromatograms are representative of three independent experiments.*

**
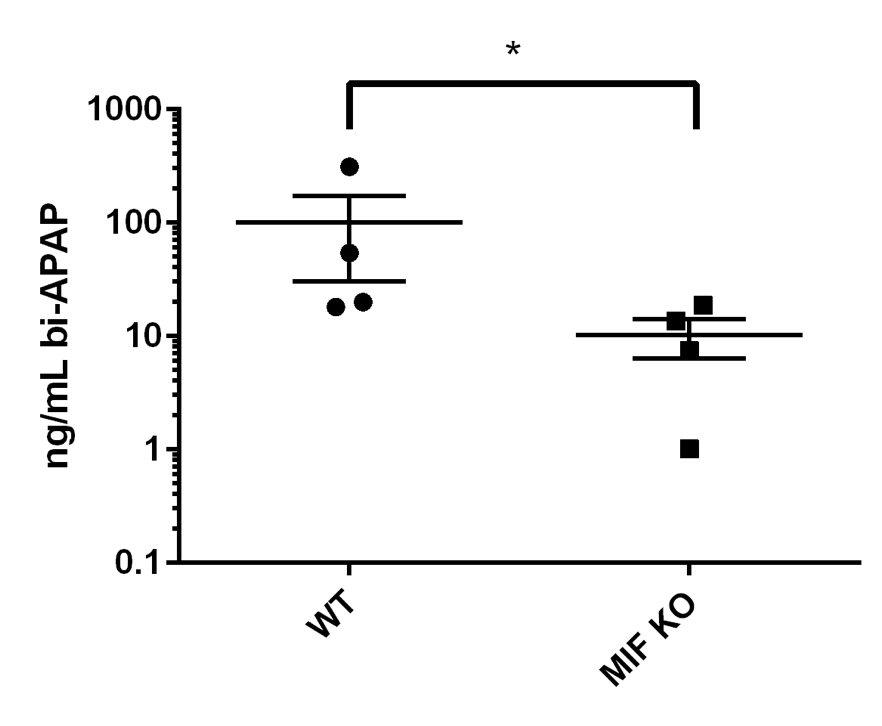
**

**Supplemental Figure 3: Bi-APAP is excreted in a MIF-dependent fashion during APAP overdose.** *C57BL/6 wild-type and MIF KO mice (n=4/group) were given 400mg/kg APAP intraperitoneally, and urine was collected and processed at 24 hours as described in Materials and Methods. Results were analyzed using a nonparametric Mann-Whitney-U test with one-tailed p-values given: *, p<0.05.*

**A**

**
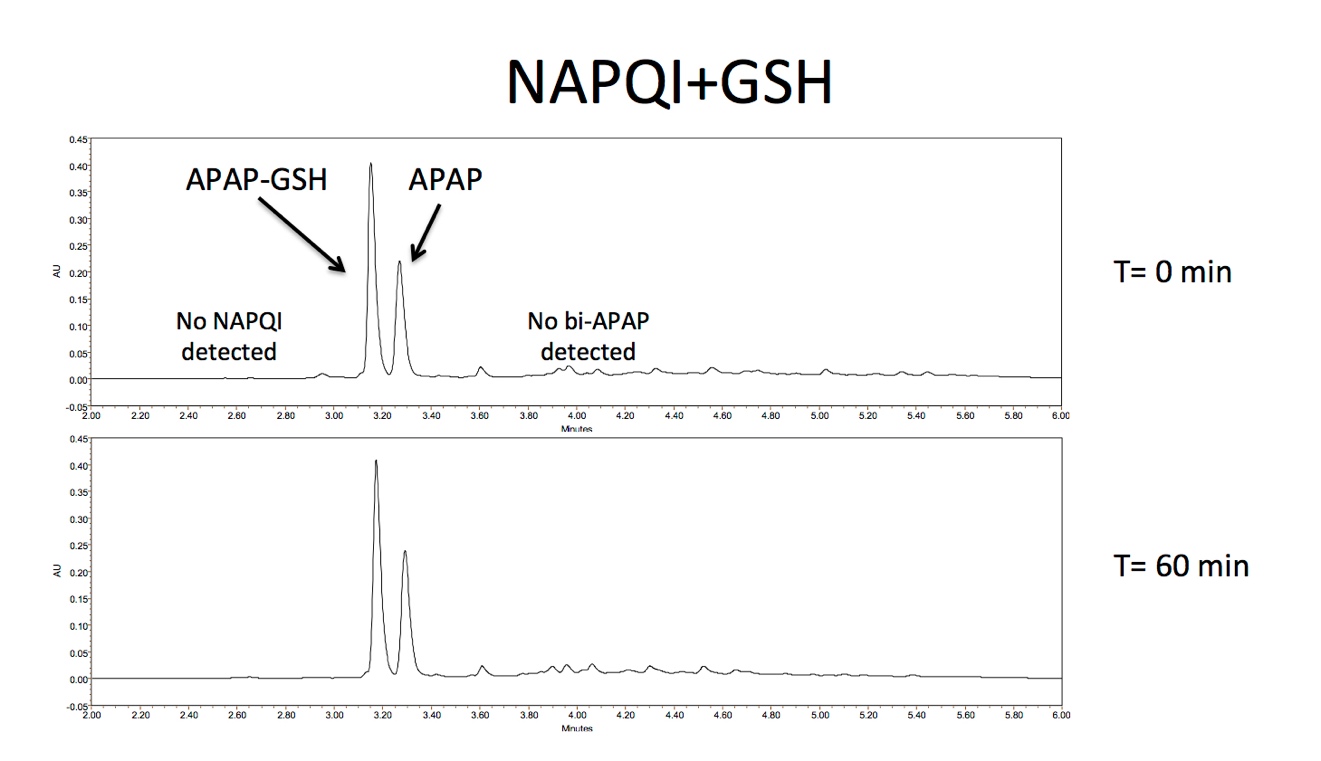
**

**B**

**
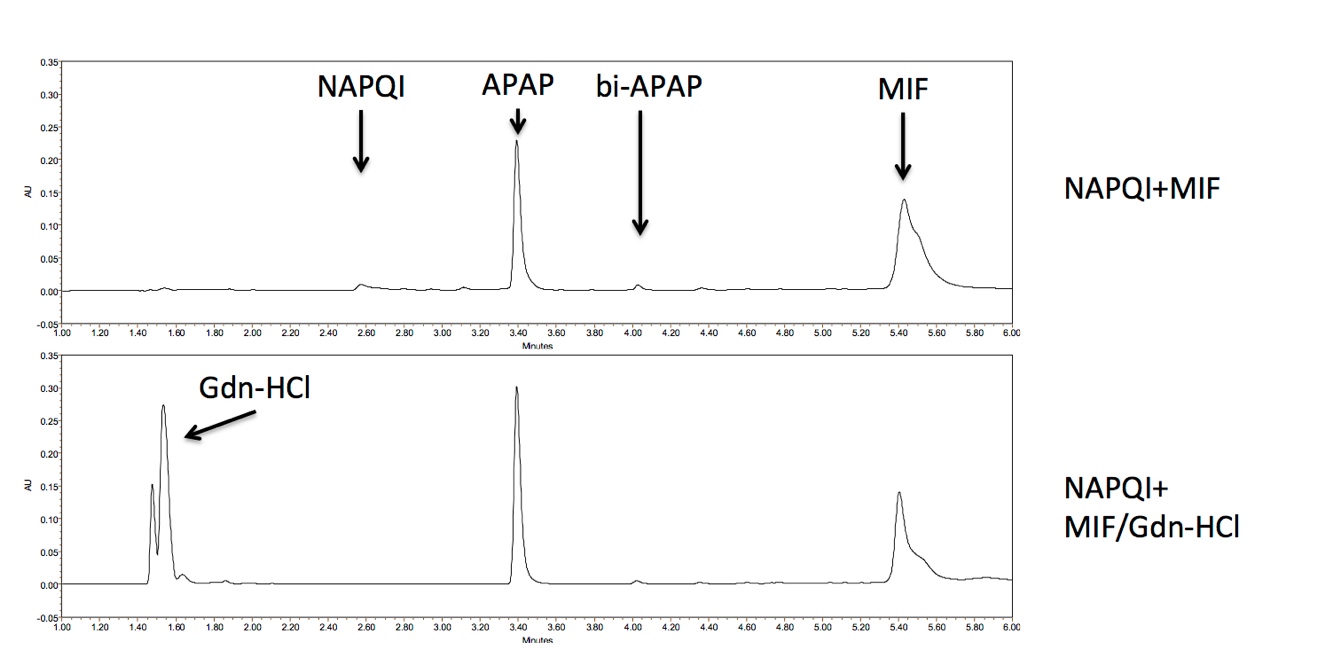
**

**Supplemental Figure 4: Effects of GSH and MIF denaturation on production of bi-APAP and APAP from MIF/NAPQI co-incubuation.** *(****A****) GSH coincubation in the NAPQI/MIF mixture does not produce peaks with chromatographic profiles consistent with bi-APAP. Representative of three independent experiments. (****B****) Gdn-HCl denatured MIF with 50% keto-enol tautomerase activity (dopachrome assay) still permits formation of bi-APAP and APAP at 60 minutes. Representative of two independent experiments. Gdn-HCl = guanidinium chloride; GSH = glutathione.*
