# Supplementary material for: Iguratimod, an allosteric inhibitor of macrophage migration inhibitory factor (MIF), prevents mortality and oxidative stress in a murine model of acetaminophen overdose
Source: Mol Med. 2024 Mar 27;30:43. doi: 10.1186/s10020-024-00803-0 (PMC10976746; doi:10.1186/s10020-024-00803-0)
Supplement: Supplementary file 2 — Supplementary Material 2 [file 10020_2024_803_MOESM2_ESM.docx]

**Left blank (title/authors/abstract/declarations removed)**

**Left blank (title/authors/abstract/declarations removed)**

**Left blank (title/authors/abstract/declarations removed)**

**BACKGROUND**

Macrophage migration inhibitory factor (MIF) is a pleiotropic cytokine expressed broadly in multiple cell types (Calandra and Roger 2003). MIF has intrinsic enzymatic activities as both a tautomerase and thiol-protein oxidoreductase (Rosengren et al. 1996; Kleemann et al. 1998). These separate and likely unrelated enzymatic sites are biologically relevant and have been used to guide the development of anti-MIF therapeutics (Pantouris et al. 2015; Thiele et al. 2015). A myriad of anti-MIF small molecules have been identified by our group and others, including compound T-614 (iguratimod), a disease-modifying anti-rheumatic drug approved for clinical use in Japan (Dios et al. 2002; Lubetsky 2002; Al-Abed 2005; Bloom et al. 2016a). Although T-614 is able to inhibit MIF keto-enol tautomerase activity as well as multiple biologically relevant functions, its binding mode and K_i_ have not been described.

Acetaminophen (APAP) overdose is the most common cause of acute liver failure in the United States and causes thousands of hospitalizations and hundreds of deaths yearly (Yoon et al. 2016; Gummin et al. 2022). When taken therapeutically APAP is mostly (~90%) subjected to Phase II metabolism in the liver, resulting in glucoronidated and sulfonated products that can be excreted renally (McGill and Jaeschke 2013). The remainder is oxidized by cytochrome P450 (CYP450) 2E1 to *N*-acetyl-*p*-benzoquinone imine (NAPQI), a reactive metabolite (Dahlin et al. 1984). At therapeutic doses of APAP, the NAPQI produced this way is further metabolized by glutathionylation to form a conjugate that can be excreted via biliary and renal routes (Wong et al. 1981). In overdose, glutathione depletion allows the metabolite NAPQI to cause cell injury and death via formation of reactive oxygen and nitrogen species and the generation of APAP-protein adducts (Blair et al. 1980; Streeter et al. 1984). The only antidote currently available is acetylcysteine, which replenishes hepatic glutathione (Prescott et al. 1977; Yoon et al. 2016). However, it has been proposed to repurpose 4-methylpyrazole (fomepizole) as a therapeutic adjunct given its ability to inhibit CYP450 2E1 and c-Jun n-terminal kinase (JNK) (Akakpo et al. 2019; Link et al. 2022).

MIF is highly expressed in hepatocytes and has been shown to promote liver injury from ethanol and carbon tetrachloride via promoting the release of inflammatory factors, such as tumor necrosis factor alpha (TNFα) and monocyte chemotactic protein 1 (MCP-1) (Barnes et al. 2013; Xie et al. 2016). MIF has also been associated with APAP-induced liver injury: MIF is released by hepatocytes in the first few hours after APAP intoxication, and acetaminophen overdose patients have higher MIF concentrations than non-overdose controls (Bourdi et al. 2002; Bloom et al. 2022). MIF-knockout (KO) animals were protected from both injury and lethality, although APAP-protein adduct formation was not different between the two backgrounds (Bourdi et al. 2002). The MIF inhibitor ISO-1 similarly attenuates inflammation and liver injury in this model (Al-Abed 2005; Ohkawara et al. 2021).

MIF-NAPQI interactions have been investigated previously. NAPQI covalently binds MIF at Pro-1, a relatively basic residue important for MIF’s tautomerase activity (Lubetsky et al. 1999; Senter et al. 2002). However, an attempt to crystallize the MIF-NAPQI complex revealed an APAP dimer, bi-acetaminophen (bi-APAP), and the electron density of the NAPQI adduct was not found (Crichlow et al. 2009). Bi-APAP has been detected in the sera and urine of APAP-intoxicated mice (Chen et al. 2008; Cheng et al. 2009) as well as culture supernatants of APAP-treated primary human hepatocytes (Jetten et al. 2015).

In this study we investigate T-614 as a MIF inhibitor with kinetic studies and X-ray crystallography, revealing a novel binding mode compared to other inhibitors of the tautomerase catalytic activity (e.g., ISO-1). We also apply T-614 to a murine model of lethal APAP overdose, with data suggesting that T-614 prevents lethality when administered as a pretreatment in experimental APAP toxicity. We assess the effects of MIF deletion and inhibition with T-614 on hepatic oxidative stress after APAP overdose. Our data confirms that MIF is involved in APAP toxicity and identifies T-614–a clinically available drug–as another therapeutic agent that can can be investigated in this disease context.

**METHODS**

*Reagents*

Acetaminophen (APAP) and guanidine hydrochloride (Gdn-HCl) were purchased from Sigma-Aldrich (St. Louis, MO). N-acetyl-p-benzoquinoneimine (NAPQI), acetaminophen dimer (bi-APAP), and acetaminophen-glutathione adduct (APAP-GSH) were purchased from Toronto Research Chemicals (Toronto, ON). NAPQI solid was maintained at -80ºC for up to six months, and a 10mM dimethyl sulfoxide (DMSO) stock solution was evacuated under nitrogen gas and stored in -80ºC for up to two weeks. Solid bi-APAP and APAP-GSH were stored at -20ºC for up to one year and solubilized in methanol for chromatography. Iguratimod (T-614) was purchased from Ontario Chemical (Guelph, ON) and stored at -20ºC. Solutions were prepared fresh in alkali (final pH 7.8) as previously described (Sawada et al. 2000) for all studies. We obtained 4-hydroxyphenylpyruvate (4-HPP) from TCI Chemicals (Portland, OR) and prepared it in pH 6.2 the day before experiments.

*Protein Expression and Purification*

Human MIF was expressed and purified following a previously established protocol (Lubetsky 2002; Parkins et al. 2023a). Briefly, a pET-11b plasmid encoding MIF was transformed into BL21(DE3) competent cells (Agilent Technologies - Cat# 200131) using the heat shock method. The cells were grown at 37˚C, in Luria Broth (LB) containing 100 μg/mL ampicillin, until the solution’s optical density at 600 nm (O.D_600_) became 0.6-0.8. Protein expression was initiated by the addition of 1 mM isopropylthio-β-galactoside (IPTG), at 37˚C, and after four hours the cells were collected and centrifuged. Cells containing MIF were lysed by sonication in 20 mM Tris HCl, 20 mM NaCl, pH 7.4 and loaded onto on Q-Sepharose and SP ion exchange chromatography columns in series. The protein did not bind to either column and eluted in the flow-through with ∼95% purity. MIF impurities were removed by size exclusion chromatography using a 16/60 Superdex 75 column (Cytiva, Marlborough, MA). The running buffer for this step was also 20 mM Tris HCl, 20 mM NaCl, pH 7.4. The final concentration of MIF was determined by the bicinchoninic acid (BCA) protein assay. Sterile recombinant MIF was maintained as 0.5-1 mg/mL stock solutions in 20 mM Tris, 20-150 mM NaCl, pH 7.4 (human) or pH 6.8 (mouse) for up to six months with no appreciable loss of activity by tautomerase assay.

*MIF Enzyme Assays and Kinetic Analyses*

Dopachrome and 4-HPP enzymatic activities of MIF were assayed as described previously (Dios et al. 2002; Parkins et al. 2023b). For dopachrome tautomerase inhibition, compounds were prepared as 100 mM stock solutions in DMSO, added to a 1.5mL cuvette (Crystalgen Inc. Commack, NY) containing 1 μg/mL MIF in PBS pH 7.4, and mixed thoroughly. Dopachrome substrate was added and the solution monitored at 475 nm for 20 seconds to measure activity. For 4-HPP tautomerase inhibition, a 30 mM stock of 4-HPP was prepared in 0.5 M ammonium acetate pH 6.2 and incubated overnight (∼16 hours) at room temperature while rocking. Then, 4-HPP was added to a 96-well microplate in a gradient of 0-2 mM final concentration, followed by the borate solution (pH 6.2) at a working concentration of 0.42 M. Inhibitor was added at a final concentration ranging from 0-50 µM in 1% DMSO. The reaction was then initiated by the addition of MIF at a final concentration of 50 nM, bringing the total well volume to 150 µL. Conversion of keto-HPP to enol-HPP was measured through the formation of the enol-HPP/borate complex (ε_306_ = 11400 M^−1^ cm^−1^) at 306 nm. Readings were taken every 10 seconds for a total period of 180 seconds using a Tecan Infinite M-Plex microplate reader (TECAN). The experiments were carried out in triplicate and the data was analyzed in GraphPad Prism 9.

*Crystallization of MIF-T-614 complex and structure determination*

Crystallization of MIF-T-614 complex was performed by the vapor diffusion method in 24-well hanging drop trays as reported previously (Pantouris et al. 2014). Freshly purified MIF (∼18 mg/mL) was mixed with T-614 at 1:3 protein-inhibitor molar ratio and incubated overnight at 4˚C. The next day, after removing precipitation by centrifugation, the MIF-T-614 complex was mixed with the well solution containing 20 mM Tris HCl pH 7.5, 2 M (NH_4_)_2_SO_4_, and 3% 2-propanol. Protein and well solutions were mixed at different volume ratios, retaining the total drop volume at 4 μL. Crystallization trays were stored at 20˚C and checked on a regular basis. Crystals were formed and grew to their full size within two weeks. For diffraction screening and data set collection, crystals were flash frozen in the mother liquor enriched with 28% glycerol (cryoprotectant). A complete data set was collected at the Macromolecular Crystallographic Facility of Yale School of Medicine using Rigaku Pilatus 200K Detector with a Rigaku 007 rotating copper anode X-ray generator (wavelength=1.5418 Å) at a temperature of 100 K. Integration and scaling was performed in HKL-2000 (Otwinowski and Minor 1997), while the solution was obtained by molecular replacement using PHASER (McCoy et al. 2007). The initial model of MIF-T-614 complex was refined using Refmac (Winn et al. 2003) and COOT (Emsley et al. 2010) and visualized with PyMOL (Delano 2002). The supporting files for T-614 (PDB and CIF) were produced by PRODRG (Schüttelkopf and Aalten 2004). Superposition of the MIF-T-614 crystal onto the corresponding structure of wild-type MIF (WT MIF) (PDB entry:3DJH) was performed in SUPERPOSE, a CCP4 supported program (Winn et al. 2011). The crystallographic table of MIF-T-614 is provided in **Suppl. Table 1** and the structure was deposited in PDB under the accession code 8SPN.

*Animal Experiments*

The Institutional Animal Care and Use Committee at the Feinstein Institute for Medical Research reviewed and approved all animal protocols prior to initiation of experiments. Male C57BL/6 NCr mice were purchased from Charles River Laboratories (Stone Ridge, NY) and acclimated in our facility for at least seven days. MIF KO animals were maintained on a C57BL/6 NCr background from the source colony described by Bozza and colleagues (Bozza et al. 1999). All animals were maintained on PicoLab Rodent Diet 20 from LabDiet (St. Louis, MO) and used between the ages of 8-12 weeks. Death was not an endpoint, and when animals were deemed moribund, they were euthanized.

*APAP-Induced Hepatotoxicity*

For all experiments, animals were fasted for 16 hours by transferring them to clean cages that never contained food prior to dosing with APAP. APAP was administered by intraperitoneal injection of a 15 mg/mL solution in warm 0.9% saline (Hospira, Lake Forest, IL). Injection volumes were adjusted to mouse weight. Volumes up to 0.8 mL were well tolerated. After APAP administration, food was provided *ad libitum*. For survival experiments, animals were dosed with 420 mg/kg APAP and monitored for two weeks; when applicable, T-614 (20 mg/kg) was administered intraperitoneally 1 hour pre-APAP, 6 hours post-APAP, and once daily in the morning for four days afterward. For acute toxicity experiments, animals were given a non-lethal dose of APAP (300 mg/kg) and euthanized at four hours post-APAP by CO_2_ asphyxiation with cervical dislocation. Blood and liver were harvested for analysis. When applicable, T-614 (20mg/kg) was administered intraperitoneally 1 hour pre-APAP and 1 hour post-APAP. Blood was collected by cardiac puncture and allowed to clot for 20 minutes at room temperature and 20 minutes at 4ºC. Sera were isolated by centrifugation at 300xg for 10 minutes and stored at -20ºC for further analysis. Livers were mobilized and divided into major lobes, and median lobes were processed for H_2_O_2_ studies. For urine collection experiments, animals were dosed with 400 mg/kg APAP and left undisturbed for 24 hours, after which urine was collected by the “single-animal method” as previously described (Kurien and Scofield 1999). Collections were done on parafilm, and 2-3 collections were performed within a 1-hour period. Urine proteins were denatured by 1:1 addition of acetonitrile (Fisher), flash frozen in liquid nitrogen, and lyophilized to dryness. Dried samples were stored at -20ºC until analysis. Liver samples subjected to HPLC analysis were similarly denatured, flash frozen, lyophilized, and stored at -20ºC until analysis.

Serum levels of alanine transaminase (ALT) were determined using kits purchased from Bioo Scientific (Austin, TX). Serum concentrations of cytokines were determined using DuoSet ELISA kits from R&D Systems (Minneapolis, MN). Liver H_2_O_2_ content was determined using a Hydrogen Peroxide Assay Kit from Abcam (Cambridge, United Kingdom). For this application, livers were homogenized by 15 passes in a Dounce homogenizer and deproteinized with perchloric acid as per the manufacturer’s recommendation, and assays were performed on the same day as tissue isolation.

*Statistics*

In this study, we attempted to display individual data point values when feasible. Raw data and calculations can be made available on request. Statistical methods are cited when applicable, and calculations were done using GraphPad Prism (San Diego, CA).

**RESULTS**

**T-614 is a non-competitive inhibitor of MIF with a novel binding motif.** To determine the K_i_ value of T-614, we performed a kinetic experiment using 4-HPP as the model substrate (**Fig. 1A-B**). The Michaelis-Menten (**Fig. 1C**) and Lineweaver-Burk plots (**Fig. 1D**) demonstrate that T614 binds MIF as a non-competitive inhibitor with a K_i_ value of 16 μM, which is on a similar scale to the IC_50_ of 6.8 μM (human MIF) with dopachrome substrate as described previously (Bloom et al. 2016a), and the IC_50_ of 5.2 μM we have observed in murine MIF. Of note, the serum C_max_ of T-614 in humans after multiple twice-daily doses of 25 mg tablets is 4.3-4.6 μM depending on age group (Careram (R) [package insert] 2013).

**Figure 1**. **Kinetic analysis of MIF-T-614 complex**. *Chemical structures of (****A****) the MIF inhibitor T-614 and (****B****) the substrate 4-HPP for MIF-induced keto-enol tautomerization. (****C****) The Michaelis-Menten and (****D****) Lineweaver-Burk plots demonstrate that the inhibitor binds MIF in a non-competitive fashion. The inhibition potency of T-614 against MIF’s keto-enol tautomerase activity was investigated at concentrations ranging from 0-50 µM and yielded an inhibition constant (K_i_) of 16 µM. Data is expressed as mean±SD from experiments done in triplicate (n=3). T614 = iguratimod.*

To further explore the binding motif of this inhibitor, we performed MIF-T-614 co-crystallization experiments. The electron density map of T-614 was clearly observed in one of the three MIF subunits (**Suppl. Table 1, Suppl. Fig. 1**). Alignment of MIF-T-614 crystal structure onto the corresponding structure of WT MIF demonstrated high superposition agreement with a root-mean-square deviation (RMSD) value of 0.17 Å, indicating that binding of T-614 did not cause any major conformational changes (**Fig. 2A**).

**Figure 2**. **Crystallographic analysis of the MIF-T-614 complex**. *(****A****) Alignment of WT MIF (grey cartoon) onto MIF-T-614 (blue cartoon) demonstrates high superposition agreement between the two crystal structures. (****B****) Analysis of the binding motif of T-614 (orange sticks) showed that the inhibitor predominantly binds on the surface of MIF, blocking the active site opening. For reference, the position of the catalytically active Pro-1 (blue sticks) is provided. (****C)*** *Only a small chemical moiety of bound T-614 penetrates the active site. The active site of MIF is illustrated as a dark grey shadow. (****D****) Stabilizing forces between T-614 and MIF. T-614 forms hydrogen bonds (Lys-32 and Ile-64, black dashed lines, residues shown as sticks), van der Waals interactions (Pro-1, Lys-32, Tyr-36, Ser-63, Ile-64 Trp-108A and Phe-113 from subunit C and Tyr-95 from subunit B, black lines), and Pi-Pi interactions (Tyr-36, red dashed line) with MIF residues. T614 = iguratimod.*

To further elucidate the effect of T-614 binding, we performed structural analysis around the inhibitor’s binding site. Surprisingly, T-614 primarily binds on the surface of MIF blocking the catalytic pocket of the tautomerase active site (**Fig. 2B**). The mesyl group of T-614 is the only chemical moiety that penetrates the pocket (**Fig. 2C**). Apart from the van der Waals interactions with Pro-1, Ser-63, Ile-64 from chain C and Tyr-95 from chain B, the mesyl group also forms a hydrogen bond with Ile-64 at 2.9 Å (**Fig. 2D**). Another hydrogen bond at 3.1 Å was noted between T-614 and Lys-32, while Tyr-36 is located less than 4 Å from T-614 and forms Pi-Pi interactions with the inhibitor (**Fig. 2D**).

**T-614 pretreatment attenuates lethality in a model of lethal APAP overdose.** We observed that animals given a non-lethal overdose of APAP (300 mg/kg) showed a peak in MIF concentrations roughly coincident with ALT, and preceding elevations in another pro-inflammatory cytokine, TNFα (**Fig. 3A**), which is consistent with the findings of Bourdi and colleagues (Bourdi et al. 2002). Based on this we designed an experiment comparing T-614 treatment prior to and after this peak. Control animals dosed with 420 mg/kg intraperitoneal APAP had 100% mortality. T-614 treatment initiated prior to APAP overdose yielded a statistically significant improvement in survival (p<0.01 by log-rank test), but treatment initiated 6 hours after overdose was not significantly different from control (**Fig. 3B-C**).

**Figure 3: Early administration of T-614 prevents lethality in APAP overdose.** *(****A****) Serum concentrations of MIF peak with ALT, and prior to TNFα. C57BL/6 mice (n=3-4/group) were given 300mg/kg APAP intraperitoneally and euthanized at indicated time points for blood collection by cardiac puncture. Results are shown mean±SD. (****B-C****) C57BL/6 wild-type mice (n=10/group) were given 420mg/kg APAP intraperitoneally in addition to T-614 vehicle (control) or T-614 initiated as pretreatment (1h pre) or post-treatment only (6h post) as described in Materials and Methods, and were monitored for two weeks; methods and time course are summarized in (****B****), and results are shown in (****C****). Death was not an endpoint for this experiment. A log-rank test was used to determine statistical significance: Control vs. T-614 (1h pre), **p<0.01; Control vs. T-614 (6h post), p>0.05. T-614 = iguratimod.*

**T-614 prevents oxidative stress in the APAP model.** Cellular toxicity in APAP overdose is partly mediated by reactive oxygen species (ROS) generated after the production of NAPQI in hepatocytes. Previous studies have shown that reactions between MIF and NAPQI can generate an MIF-NAPQI adduct or an APAP dimer, bi-APAP (Senter et al. 2002; Crichlow et al. 2009). We have also observed that bi-APAP and APAP can be generated when these molecules are co-incubated, and excretion of bi-APAP in murine APAP toxicity appears to be MIF dependent (**Suppl. Fig. 2-4**). Given the possibility that MIF could be involved in oxidation-reduction processes during APAP toxicity, we wanted to pursue a potential effect of MIF on oxidative stress in this model (**Fig. 4A**). Hydrogen peroxide is a byproduct of ROS generation *in vivo*, and can be directly measured as a surrogate of oxidative stress in tissue. T-614-treated WT mice given a non-lethal overdose of APAP had significantly less H_2_O_2_ detected in hepatic tissue (**Fig. 4B**). MIF KO animals treated under the same conditions showed a similarly significant decrease in hepatic tissue H_2_O_2_ (p<0.05 for non-T-614 treated WT versus MIF KO), and no additional effect of T-614 was seen in MIF KO animals (**Fig. 4C**), suggesting T-614 effects on this readout may be MIF-specific.

**Figure 4: T-614 prevents oxidative stress in APAP overdose***. (****A****) Experimental summary.* *(****B****) C57BL/6 wild-type and (****C****) MIF KO mice (n=4-7/group) were given 300/kg APAP intraperitoneally in addition to doses of intraperitoneal T-614 or vehicle (1 hour pre- and post-APAP) and euthanized at 4 hours for tissue collection and H_2_O_2_ assay as described in Materials and Methods. Results are shown as mean±SD and were analyzed using unpaired t-tests with one-tailed p-values given: *, p<0.05. T614 = iguratimod.*

**DISCUSSION**

T-614 is an effective disease-modifying antirheumatic drug currently in clinical use in Japan. Initial studies suggested its mechanism of action may operate via interactions with cyclooxygenase-2 or effects on translocation of nuclear factor kappa B. Our group recently established that it functions as a MIF inhibitor as well, and this bioactivity could explain some of its anti-inflammatory actions (Tanaka et al. 1995; Aikawa et al. 2002; Bloom et al. 2016a). Given MIF is a broadly expressed cytokine with putative roles in multiple disease states, the application of a clinically available drug that can modulate MIF activity could have important implications, and there is a need to explore the therapeutic potential of repurposing this drug (Bozza et al. 1995; Onodera et al. 2000; Jong et al. 2001). Our findings here confirm that T-614 not only interacts with the MIF trimer, but also acts as an allosteric inhibitor with a novel binding mode. Tautomerase inhibition is mediated by steric blockade of the active site. Occupancy in this region may also disrupt MIF interactions with its putative receptor CD74, and subsequent pro-inflammatory effects, although further studies would be needed to confirm this (Leng et al. 2003; Pantouris et al. 2014). The structural interactions between MIF and CD74 have been mapped previously, and T-614 interacts with multiple residues shown to have a key role in the MIF-induced activation of CD74 (Pantouris et al. 2015).

MIF has previously been studied in APAP toxicity, and the pathophysiology of its interaction with NAPQI has been poorly elucidated. Coincubation of NAPQI and MIF appeared to produce a MIF-NAPQI adduct at the nucleophilic Pro-1 based on mass spectrometry analysis, but a P1S mutant MIF did not generate an adduct (Senter et al. 2002). A crystallography study did not show this adduct, and instead revealed a noncovalently bound molecule of bi-APAP at the MIF active site (Crichlow et al. 2009). Our comparatively simple chromatography experiments similarly revealed a mixture of products after MIF-NAPQI co-incubation, and it seems likely that both the MIF-NAPQI adduct and bi-APAP can be generated in this reaction, among other potential products. Bi-APAP may not be a direct enzymatic product, but rather a side product formed by comproportionation between NAPQI and residual APAP, which has been described previously (Potter and Hinson 1986). Studies of relative affinity of NAPQI for APAP and glutathione suggest that dimer formation would be inhibited in the presence of glutathione, which has a much higher second-order rate constant for NAPQI at pH 6.5 (1x10^4^M^-1^sec^-1^) than APAP (33M^-1^sec^-1^) (Potter and Hinson 1986). In our experimental model of NAPQI incubation, bi-APAP peaks could not be detected when glutathione was present (**Suppl. Fig. 4A**). It is possible that in the absence of glutathione–for example, during the glutathione depletion observed in APAP overdose–dimer formation is permitted via comproportionation reactions between APAP and NAPQI that produce N-acetyl-p-benzosemiquinone imine (NAPSQI), which can react further to produce APAP polymers such as bi-APAP. MIF appears to facilitate or permit this process, since a MIF KO animal excreted significantly less bi-APAP, although whether this effect is attributable to a direct interaction between MIF and NAPQI or another mechanism is unclear. Partial ablation of tautomerase activity by denaturation of MIF with Gdn-HCl did not affect the production of bi-APAP after coincubation (**Suppl. Fig. 4B**). Of note, MIF can also act as a thiol-protein oxidoreductase based on a CXXC motif around Cys-56 and Cys-59, and this motif and activity appear important for MIF interactions with intracellular binding partners (Kleemann et al. 1998; Bloom et al. 2016b); it is unknown at this time whether this motif influences MIF effects on APAP toxicity.

Our findings suggest that MIF inhibition with T-614 can prevent APAP toxicity: T-614-treated animals had less hydrogen peroxide present in liver tissue, and no additional effect of T-614 was seen in MIF KO mice. As we do not document baseline hydrogen peroxide levels in WT mice, it is unclear if T-614 treatment restored these levels to an untreated baseline. It is possible that the extent of protection from MIF genetic deletion was such that any additional non-MIF-related protective effect of T-614 would not be observable. Additionally, non-MIF related effects are possible, such as T-614 inhibition of CYP450. However, our results suggest that the effects of T-614 could be MIF-specific. Based on our observation that lethality in the APAP model was only attenuated by early administration of T-614, MIF’s pathologic role in murine APAP toxicity is expected to occur earlier than six hours after APAP administration. This accords with observations by our group and others of early peaks in serum MIF concentrations after APAP overdose (Bourdi et al. 2002). Of note, our experiments demonstrated a relatively milder level of toxicity (based on lethality and ALT release) per APAP mg/kg dosing compared to Bourdi’s study or more recent studies using this mouse background (Duan et al. 2016). Although our experimental conditions differed slightly from these studies, this is an important limitation to our findings.

Unlike conventional cytokines, MIF is expressed at baseline and is stored in cytoplasmic pools for release in response to stimuli; humans also have detectable MIF in plasma at baseline (Bacher et al. 1997; Kudrin et al. 2006). The early phase of APAP toxicity involves NAPQI generation and cellular toxicity via generation of reactive species and protein adduct formation. Subsequent sterile inflammation is marked by the release of conventional cytokines such as TNFα, which we observed beginning at 4 hours. Since we only compared treatment with T-614 prior to APAP and 6 hours after APAP administration, we are unable to determine by time course alone whether the benefits of MIF inhibition occurred during the NAPQI/reactive products phase or the inflammatory phase, which is a limitation of our study. Other important limitations of our study include a lack of liver histology to confirm liver injury, and a lack of ALT measurement at all doses and time points in our models. In the absence of this information, we cannot recommend clinical application of T-614 in APAP toxicity as yet. Future studies could help resolve this question by testing anti-MIF therapy at more acute time points (before 6 hours) with more extensive monitoring of liver histology and transaminases, or probing other biomarkers of APAP toxicity (Davern et al. 2006); comparison with other known and proposed antidotes for APAP toxicity (acetylcysteine, fomepizole) would also be useful. Of interest, prior studies of MIF KO showed no effect on APAP-protein adducts, but inhibition with ISO-1 resulted in increased measured glutathione stores in liver tissue from APAP-intoxicated mice, which would seem to be contradictory (Bourdi et al. 2002; Ohkawara et al. 2021). Early studies of MIF suggested possible enzymatic activity as a glutathione-S-transferase, but subsequent experiments did not support this (Blocki et al. 1992; Pearson 1994; Mühlhahn et al. 1996; Swope et al. 1998).

Prior investigations have suggested that JNK1 and JNK2 are mechanistically important in the development of hepatocellular toxicity from APAP, and the alcohol dehydrogenase and JNK inhibitor 4-methylpyrazole (fomepizole) is under investigation as potential target for drug repurposing in APAP toxicity (Gunawan et al. 2006; Akakpo et al. 2019). Fomepizole was able to provide hepatoprotection by multiple readouts when dosed as either a pre-treatment or 90 minutes post-APAP (Akakpo et al. 2018, 2019). MIF is known to influence JNK phosphorylation, although its effects appear to be pleiotropic based on cell type: recombinant MIF stimulated JNK phosphorylation in fibroblasts and T-cell lines but attenuated it in murine hepatocytes and HepG2 cells under lipemic conditions (Lue et al. 2011; Cui et al. 2022). The MIF-JNK relationship may affect or drive MIF-mediated effects in APAP toxicity, and future studies are needed to investigate this.

**CONCLUSIONS**

We have found that T-614 is an allosteric inhibitor of MIF with a unique binding mode outside the catalytic pocket of the tautomerase active site. Our work also establishes that it can prevent oxidative stress and lethality in a murine model of APAP hepatotoxicity, suggesting that T-614 should be investigated further to determine its therapeutic potential in APAP hepatotoxicity. Further studies are needed to elucidate the molecular mechanisms of MIF and MIF inhibition in this disease process.

**LIST OF ABBREVIATIONS**

4-HPP 4-hydroxyphenylpyruvate

ALT Alanine transaminase

APAP Acetaminophen

BCA bicinchoninic acid

Bi-APAP bi-acetaminophen

CYP450 Cytochrome P450

Cys Cysteine

DMSO Dimethyl sulfoxide

Gdn Guanidinium

GSH Glutathione

HPLC High performance liquid chromatography

IPTG isopropylthio-β-galactoside

JNK c-Jun n-terminal kinase

KO Knockout

Ki Inhibition constant

LB Luria Broth

MCP-1 Monocyte chemotactic protein 1

MIF Macrophage migration inhibitory factor

NAPQI N-acetyl-p-benzoquinoneimine

NAPSQI N-acetyl-p-benzosemiquinoneimine

PBS Phosphate-buffered saline

ROS Reactive oxygen species

T-614 Iguratimod

TBS Tris-buffered saline

TNFα Tumor necrosis factor alpha

WT Wild type

**DECLARATIONS**

**Ethics approval and consent to participate:** All animal experiments were performed in accordance to the National Institute of Health (NIH) guidelines and under protocols approved by the IACUC of the Feinstein Institute for Medical Research.

**Consent for publication:** Not applicable.

**Availability of data and materials:** The crystallographic structure of MIF-T-614 was deposited in Research Collabatory for Structural Bioinformatics Protein Data Bank (RCSB PDB) under the accession code 8SPN.

**Competing interests:** JB and YA are co-holders of a patent for the use of T-614 as an inhibitor of MIF. YA is an inventor or co-inventor for multiple other small molecule inhibitors of MIF. MH, BA, and YA are current staff at the Feinstein Institutes for Medical Research. JB received a stipend as a MD/PhD candidate in the Zucker School of Medicine at Hofstra/Northwell Health, and his PhD studies were completed at the Feinstein Institutes for Medical Research. The authors have no other potential conflicts of interest to declare.

**Funding:** This work was supported by funding from the Feinstein Institutes for Medical Research. This work was also partially supported by start-up funds from University of the Pacific (author GP).

**Authors' contributions:** GP, RM, and EJL collaborated to produce MIF kinetic and crystallography data. AP performed the kinetics experiments. JB, MH, BA, LM, and YAA collaborated on all animal studies and HPLC experiments. JB and GP collaborated on the structure and content of the manuscript. EJL and YAA conceived the underlying ideas of the project, oversaw the preparation of the manuscript, and gave final approval of the version to be published.

**Acknowledgements:** We would like to thank Hunter College Mass Spectrometry for their assistance in analyzing urine samples. Hunter Mass Spectrometry is supported by the City University of New York (CUNY), the National Science Foundation (NSF), and the National Institute on Minority Health and Health Disparities (NIMHD) of the National Institutes of Health (NIH).

**Authors' information (optional):** N/A

**BIBLIOGRAPHY**

Aikawa Y, Yamamoto M, Yamamoto T, Morimoto K, Tanaka K. An anti-rheumatic agent T-614 inhibits NF-κB activation in LPS- and TNF-α-stimulated THP-1 cells without interfering with IκBα degradation. Inflamm Res. 2002;51:188–94.

Akakpo J, Ramachandran A, Kandel S, Ni H, Kumer S, Rumack B, et al. 4-Methylpyrazole protects against acetaminophen hepatotoxicity in mice and in primary human hepatocytes. Hum Exp Toxicol. 2018;37:1310–22.

Akakpo JY, Ramachandran A, Duan L, Schaich MA, Jaeschke MW, Freudenthal BD, et al. Delayed Treatment With 4-Methylpyrazole Protects Against Acetaminophen Hepatotoxicity in Mice by Inhibition of c-Jun n-Terminal Kinase. Toxicol Sci. 2019;170:57–68.

Al-Abed Y. ISO-1 Binding to the Tautomerase Active Site of MIF Inhibits Its Pro-inflammatory Activity and Increases Survival in Severe Sepsis. Journal of Biological Chemistry. 2005;280:36541–4.

Bacher M, Meinhardt A, Lan HY, Mu W, Metz CN, Chesney JA, et al. Migration inhibitory factor expression in experimentally induced endotoxemia. The American Journal of Pathology. 1997;150:235–46.

Barnes MA, McMullen MR, Roychowdhury S, Pisano SG, Liu X, Stavitsky AB, et al. Macrophage migration inhibitory factor contributes to ethanol‐induced liver injury by mediating cell injury, steatohepatitis, and steatosis. Hepatology. 2013;57:1980–91.

Blair IA, Boobis AR, Davies DS, Cresp TM. Paracetamol oxidation: synthesis and reactivity of N-acetyl-p-benzoquinoneimine. Tetrahedron Letters. 1980;21:4947–50.

Blocki FA, Schlievert PM, Wackett LP. Rat liver protein linking chemical and immunological detoxification systems. Nature. 1992;360:269–70.

Bloom J, Metz C, Nalawade S, Casabar J, Cheng KF, He M, et al. Identification of Iguratimod as an Inhibitor of Macrophage Migration Inhibitory Factor (MIF) with Steroid-Sparing Potential. The Journal of Biological Chemistry. 2016;291:26502-514.

Bloom J, Sun S, Al-Abed Y. MIF, a controversial cytokine: a review of structural features, challenges, and opportunities for drug development. Expert Opin Ther Tar. 2016;20:1463–75.

Bloom J, Uzamere T, Hurd Y, Manini AF. Macrophage migration inhibitory factor as a potential biomarker in acetaminophen overdose: a pilot study. Toxicol Commun. 2022;6:1–5.

Bourdi M, Reilly TP, Elkahloun AG, George JW, Pohl LR. Macrophage migration inhibitory factor in drug-induced liver injury: a role in susceptibility and stress responsiveness. Biochemical and Biophysical Research Communications. 2002;294:225–30.

Bozza M, Kolakowski LF, Jenkins NA, Gilbert DJ, Copeland NG, David JR, et al. Structural characterization and chromosomal location of the mouse macrophage migration inhibitory factor gene and pseudogenes. Genomics. 1995;27:412–9.

Bozza M, Satoskar AR, Lin G, Lu B, Humbles AA, Gerard C, et al. Targeted Disruption of Migration Inhibitory Factor Gene Reveals Its Critical Role in Sepsis. J Exp Medicine. 1999;189:341–6.

Calandra T, Roger T. Macrophage migration inhibitory factor: a regulator of innate immunity. Nature Reviews Immunology. 2003;3:791–800.

Careram® Tablets [package insert]. Tokyo, Japan: Eisai Co. Ltd; 2013.

Chen C, Krausz KW, Idle JR, Gonzalez FJ. Identification of novel toxicity-associated metabolites by metabolomics and mass isotopomer analysis of acetaminophen metabolism in wild-type and Cyp2e1-null mice. The Journal of Biological Chemistry. 2008;283:4543–59.

Cheng J, Ma X, Krausz KW, Idle JR, Gonzalez FJ. Rifampicin-Activated Human Pregnane X Receptor and CYP3A4 Induction Enhance Acetaminophen-Induced Toxicity. Drug metabolism and disposition: the biological fate of chemicals. 2009;37:1611–21.

Crichlow GV, Lubetsky JB, Leng L, Bucala R, Lolis EJ. Structural and Kinetic Analyses of Macrophage Migration Inhibitory Factor Active Site Interactions. Biochemistry. 2009;48:132–9.

Cui N, Li H, Dun Y, Ripley-Gonzalez JW, You B, Li D, et al. Exercise inhibits JNK pathway activation and lipotoxicity via macrophage migration inhibitory factor in nonalcoholic fatty liver disease. Frontiers Endocrinol. 2022;13:961231.

Dahlin DC, Miwa GT, Lu AY, Nelson SD. N-acetyl-p-benzoquinone imine: a cytochrome P-450-mediated oxidation product of acetaminophen. Proceedings of the National Academy of Sciences of the United States of America. 1984;81:1327–31.

Davern TJ, James LP, Hinson JA, Polson J, Larson AM, Fontana RJ, et al. Measurement of Serum Acetaminophen–Protein Adducts in Patients With Acute Liver Failure. Gastroenterology. 2006;130:687–94.

Delano W. The PyMOL molecular graphics system. Palo Alto, CA: Delano Scientific; 2002.

Dios A, Mitchell RA, Aljabari B, Lubetsky J, O’Connor K, Liao H, et al. Inhibition of MIF Bioactivity by Rational Design of Pharmacological Inhibitors of MIF Tautomerase Activity. J Med Chem. 2002;45:2410–6.

Duan L, Davis JS, Woolbright BL, Du K, Cahkraborty M, Weemhoff J, et al. Differential susceptibility to acetaminophen-induced liver injury in sub-strains of C57BL/6 mice: 6N versus 6J. Food and Chemical Toxicology. 2016; 98: 107-118.

Emsley P, Lohkamp B, Scott WG, Cowtan K. Features and development of Coot. Acta Crystallogr Sect D Biological Crystallogr. 2010;66:486–501.

Gummin DD, Mowry JB, Beuhler MC, Spyker DA, Rivers LJ, Feldman R, et al. 2021 Annual Report of the National Poison Data System© (NPDS) from America’s Poison Centers: 39th Annual Report. Clin Toxicol. 2022;60:1381–643.

Gunawan BK, Liu Z, Han D, Hanawa N, Gaarde WA, Kaplowitz N. c-Jun N-Terminal Kinase Plays a Major Role in Murine Acetaminophen Hepatotoxicity. Gastroenterology. 2006;131:165–78.

Jetten MJA, Ruiz-Aracama A, Coonen MLJ, Claessen SM, Herwijnen MHM, Lommen A, et al. Interindividual variation in gene expression responses and metabolite formation in acetaminophen-exposed primary human hepatocytes. Archives of Toxicology. 2016;90:1103-15.

Jong YP de, Abadia-Molina AC, Satoskar AR, Clarke K, Rietdijk ST, Faubion WA, et al. Development of chronic colitis is dependent on the cytokine MIF. Nature Immunology. 2001;2:1061–6.

Kleemann R, Kapurniotu A, Frank RW, Gessner A, Mischke R, Flieger O, et al. Disulfide analysis reveals a role for macrophage migration inhibitory factor (MIF) as thiol-protein oxidoreductase. Journal of Molecular Biology. 1998;280:85–102.

Kudrin A, Scott M, Martin S, Chung CW, Donn R, McMaster A, et al. Human macrophage migration inhibitory factor: a proven immunomodulatory cytokine? The Journal of Biological Chemistry. 2006;28:29641–51.

Kurien BT, Scofield RH. Mouse urine collection using clear plastic wrap. Laboratory Animals. 1999;33:83–6.

Leng L, Metz CN, Fang Y, Xu J, Donnelly S, Baugh J, et al. MIF Signal Transduction Initiated by Binding to CD74. Journal of Experimental Medicine. 2003;197:1467–76.

Link SL, Rampon G, Osmon S, Scalzo AJ, Rumack BH. Fomepizole as an adjunct in acetylcysteine treated acetaminophen overdose patients: a case series. Clin Toxicol. 2022;60:472–7.

Lubetsky JB. The Tautomerase Active Site of Macrophage Migration Inhibitory Factor Is a Potential Target for Discovery of Novel Anti-inflammatory Agents. Journal of Biological Chemistry. 2002;277:24976–82.

Lubetsky JB, Swope M, Dealwis C, Blake P, Lolis E. Pro-1 of Macrophage Migration Inhibitory Factor Functions as a Catalytic Base in the Phenylpyruvate Tautomerase Activity. Biochemistry. 1999;38:7346–54.

Lue H, Dewor M, Leng L, Bucala R, Bernhagen J. Activation of the JNK signalling pathway by macrophage migration inhibitory factor (MIF) and dependence on CXCR4 and CD74. Cell Signal. 2011;23:135–44.

McCoy AJ, Grosse-Kunstleve RW, Adams PD, Winn MD, Storoni LC, Read RJ. Phaser crystallographic software. J Appl Crystallogr. 2007;40:658–74.

McGill MR, Jaeschke H. Metabolism and Disposition of Acetaminophen: Recent Advances in Relation to Hepatotoxicity and Diagnosis. Pharmaceutical Research. 2013;30:2174–87.

Mühlhahn P, Bernhagen J, Czisch M, Georgescu J, Renner C, Ross A, et al. NMR characterization of structure, backbone dynamics, and glutathione binding of the human macrophage migration inhibitory factor (MIF). Protein Sci. 1996;5:2095–103.

Ohkawara T, Okubo N, Maehara O, Nishihira J, Takeda H. Protective effect of ISO-1 with inhibition of RIPK3 up-regulation and neutrophilic accumulation on acetaminophen-induced liver injury in mice. Toxicology Letters. 2021;339:51–9.

Onodera S, Kaneda K, Mizue Y, Koyama Y, Fujinaga M, Nishihira J. Macrophage migration inhibitory factor up-regulates expression of matrix metalloproteinases in synovial fibroblasts of rheumatoid arthritis. The Journal of Biological Chemistry. 2000;275:444–50.

Otwinowski Z, Minor W. [20] Processing of X-ray diffraction data collected in oscillation mode. Methods Enzymol. 1997;276:307–26.

Pantouris G, Rajasekaran D, Garcia AB, Ruiz VG, Leng L, Jorgensen WL, et al. Crystallographic and Receptor Binding Characterization of Plasmodium falciparum Macrophage Migration Inhibitory Factor Complexed to Two Potent Inhibitors. J Med Chem. 2014;57:8652–6.

Pantouris G, Syed MA, Fan C, Rajasekaran D, Cho TY, Jr EMR, et al. An Analysis of MIF Structural Features that Control Functional Activation of CD74. Chemistry & Biology. 2015;22:1197–205.

Parkins A, Das P, Prahaladan V, Rangel VM, Xue L, Sankaran B, et al. 2,5-Pyridinedicarboxylic acid is a bioactive and highly selective inhibitor of D-dopachrome tautomerase. Structure. 2023;31:355-367.

Parkins A, Sandin SI, Knittel J, Franz AH, Ren J, Alba E de, et al. Underrepresented Impurities in 4‑Hydroxyphenylpyruvate Affect the Catalytic Activity of Multiple Enzymes. Anal Chem. 2023;95:4957–65.

Pearson WR. MIF proteins are not glutathione transferase homologs. Protein Science. 1994; 3:525-7.

Potter DW, Hinson JA. Reactions of N-acetyl-p-benzoquinone imine with reduced glutathione, acetaminophen, and NADPH. Molecular Pharmacology. 1986;30:33–41.

Prescott LF, Park J, Ballantyne A, Adriaenssens P, Proudfoot AT. Treatment of paracetamol (acetaminophen) poisoning with N-acetylcysteine. Lancet (London, England). 1977;2:432–4.

Rosengren E, Bucala R, Aman P, Jacobsson L, Odh G, Metz CN, et al. The immunoregulatory mediator macrophage migration inhibitory factor (MIF) catalyzes a tautomerization reaction. Molecular Medicine (Cambridge, Mass). 1996;2:143–9.

Sawada T, Hashimoto S, Tohma S, Nishioka Y. Inhibition of L-leucine methyl ester mediated killing of THP-1, a human monocytic cell line, by a new anti-inflammatory drug, T614. Immunopharmacology. 2000;49:285–94.

Schüttelkopf AW, Aalten DMF van. PRODRG: a tool for high-throughput crystallography of protein–ligand complexes. Acta Crystallogr Sect D Biological Crystallogr. 2004;60:1355–63.

Senter PD, Al-Abed Y, Metz CN, Benigni F, Mitchell RA, Chesney J, et al. Inhibition of macrophage migration inhibitory factor (MIF) tautomerase and biological activities by acetaminophen metabolites. Proceedings of the National Academy of Sciences of the United States of America. 2002;99:144–9.

Streeter AJ, Dahlin DC, Nelson SD, Baillie TA. The covalent binding of acetaminophen to protein. Evidence for cysteine residues as major sites of arylation in vitro. Chemico-biological Interactions. 1984;48:349–66.

Swope MD, Sun HW, Klockow B, Blake P, Lolis E. Macrophage migration inhibitory factor interactions with glutathione and S-hexylglutathione. The Journal of Biological Chemistry. 1998 Jun;273:14877–84.

Tanaka K, Kawasaki H, Kurata K, Aikawa Y, Tsukamoto Y, Inaba T. T-614, a novel antirheumatic drug, inhibits both the activity and induction of cyclooxygenase-2 (COX-2) in cultured fibroblasts. Japanese Journal of Pharmacology. 1995;67:305–14.

Thiele M, Kerschbaumer RJ, Tam FWK, Völkel D, Douillard P, Schinagl A, et al. Selective Targeting of a Disease-Related Conformational Isoform of Macrophage Migration Inhibitory Factor Ameliorates Inflammatory Conditions. The Journal of Immunology. 2015;195:2343–52.

Winn MD, Ballard CC, Cowtan KD, Dodson EJ, Emsley P, Evans PR, et al. Overview of the CCP4 suite and current developments. Acta Crystallogr Sect D. 2011;67:235–42.

Winn MD, Murshudov GN, Papiz MZ. Macromolecular TLS Refinement in REFMAC at Moderate Resolutions. Methods Enzymol. 2003;374:300–21.

Wong LT, Whitehouse LW, Solomonraj G, Paul CJ. Pathways of disposition of acetaminophen conjugates in the mouse. Toxicol Lett. 1981;9:145–51.

Xie J, Yang L, Tian L, Li W, Yang L, Li L. Macrophage Migration Inhibitor Factor Upregulates MCP-1 Expression in an Autocrine Manner in Hepatocytes during Acute Mouse Liver Injury. Scientific Reports. 2016; 6:27665.

Yoon E, Babar A, Choudhary M, Kutner M, Pyrsopoulos N. Acetaminophen-Induced Hepatotoxicity: a Comprehensive Update. Journal of Clinical and Translational Hepatology. 2016;4:131–42.
